# Supplementary material for: Deciphering “the language of nature”: A transformer-based language model for deleterious mutations in proteins
Source: Innovation (Camb). 2023 Jul 27;4(5):100487. doi: 10.1016/j.xinn.2023.100487 (PMC10448337; doi:10.1016/j.xinn.2023.100487)
Supplement: Document S1. Supplemental methods, Figures S1–S7, and Tables S1–S3 and S5 [file mmc1.pdf]

The Innovation, Volume *4*

Supplemental Information

Deciphering “the language of nature”: A transformer-based language model for deleterious mutations in proteins

Theodore T. Jiang, Li Fang, and Kai Wang

The Innovation, Volume ■ ■

Supplemental Information

Deciphering "the language of nature": A transformer-based language model for deleterious mutations in proteins

Theodore T. Jiang, Li Fang, and Kai Wang

Supplemental Methods

1. Data Preparation

1.1 Pretraining data generation

We pretrained MutFormer on human reference protein sequences (all isoforms) and protein sequences caused by non-synonymous SNVs with > 1% population frequency in the gnomAD database.[1] The 1% threshold was used to ensure that this collection of examples contained a higher percentage of truly benign variants since it is important for the model to learn the “syntax” of the language from “correct” examples during pretraining. The total number of protein sequences used during pretraining was 128,670. The maximum input length of MutFormer was set to 1024, where protein sequences longer than 1024 are cut into non-overlapping segments starting with the first residue (i.e. the first segment contains residues 1-1024). Before cutting, a “B” letter was added to the beginning of a sequence, and a “J” letter was added to the end of a sequence so that the true start and end of a protein sequence were also indicated (“B” and “J” are not included in the current biological amino acid alphabet). During cutting, all segments were retained, except for segments less than 50 amino acids long, which were discarded. In total, we accumulated 150,533 training data points from these 128,670 protein sequences, of which 86,213 were left intact when compared toc their original protein sequence (with both “B” and “J” tokens), 23,857 contained a “B” token but not a “J” token, 21,672 contained a “J” token but not a “B” token, 18,791 contained neither a “B” nor “J” token, and 2,561 original protein sequences did not have its tail end represented (because the tail end of the sequence was less than 50 amino acids long).

1.2 Training and evaluation data generation for variant deleteriousness prediction

We obtained 84K manually annotated pathogenic missense SNVs from the Human Gene Mutation Database (HGMD, version 2016).[2] We combined this set with SNPs from the gnomAD database[1] with allele frequency >0.1%, the vast majority of which are assumed to be benign. Although a commonly used allele frequency threshold for benign variants is 1%, we used 0.1% instead, in order to achieve 1) the exclusion of pretraining data from the fine-tuning data (MutFormer’s pretraining data utilized the threshold of 1%); 2) the approximate balancing of the number of benign and deleterious/pathogenic examples in the dataset. Any mutations that appeared in both the HGMD database and benign set were removed, and from the remaining data, all the mutations present in the original pretraining data (i.e., >1% in gnomAD) were also eliminated. Within the training data, mutated sequences were obtained by mutating a reference protein sequence (using ANNOVAR) based on a nucleotide substitution specified by each variant in the dataset. In situations where the reference sequence residue did not match the reported reference residue by the mutation, the example was discarded. The final benign set contained 61K variants. The combination of the deleterious set and the benign set was randomly split into a training set and an independent validation set. The independent validation set was then isolated from the training set by reference sequence: to prevent memorization from the training set to the validation set, mutations were deleted from the training set if the mutated sequence or the mutation’s reference sequence was present in the validation set. The independent validation set contains 5,282 benign variants and 3,145 deleterious variants. Note that this independent validation set, despite its independent selection, is still prone to bias because of its similarity with the training set. For this reason, this set is only used to internally compare the performance of MutFormer models trained within this study, and separate testing sets compiled from various different sources are used for the comparison of MutFormer with other existing methods of deleteriousness prediction.

2. Auxiliary Tests

2.1 Analyzing bias present in MutFormer’s fine-tuning data

To gauge the amount of bias present in the resulting fine-tuning dataset, distributions of positive and negative examples per protein reference id are displayed in Figure S4A. However, since certain proteins are naturally

more sensitive to mutations than others, in order to appropriately interpret the positive and negative example distributions in the context of the human population, we graphed the probability of loss of function intolerance (pLI) score, obtained from GnomAD data for each corresponding protein ID in Figure S4B. From the two graphs, we see that protein IDs with relatively low pLI scores tend to have less deleterious representation and more benign representation in our fine-tuning dataset, suggesting that there is little bias introduced.

2.2 Ablation Study on MutFormer's Use of External Predictions

In order to assess the extent of MutFormer's use of each external prediction during pathogenicity prediction, we performed an ablation study for our best-performing model, $MutFormer_{8L}$ with integrated convolutions (full hyperparameter description in Table 3), which represents MutFormer in our comparison with existing methods. The ablation study is outlined as follows: For each external prediction, we remove it from the input while keeping all other predictions constant. The performance of the model on our independent validation set with the method removed is then recorded. As can be seen from Figure S7, the removal of MVP as a source of information for MutFormer results in a noticeably larger drop in performance than other methods. This can perhaps be explained by MVP's consistently high performance on our independent testing datasets, making it a good source of information for MutFormer. Figure S7 displays the graph resulting from this ablation study.

2.3 MutFormer Complementing Evolutionary Approaches

Due to MutFormer's primary reliance on protein sequence data analysis, we hypothesized that MutFormer would be capable of providing complementary information to existing evolutionarily based methods for variant classification. To assess this, we compared MutFormer (external prediction included) to EVE[3] using the provided scores and prediction data from EVE's database. Only examples with reported clinical significance of benign, pathogenic, likely pathogenic, or likely benign were used. We calculated the Spearman Rank Correlation between MutFormer and EVE for mutations predicted by both MutFormer EVE. We found rank correlations of 0.320 and 0. 336 (both with p values less than $1x10^{-10}$) between MutFormer and EVE_ASM and between MutFormer and EVE_BPU, respectively. This rank correlation indicates that while MutFormer is correlated with EVE, the information provided by MutFormer is not identical to that which EVE provides, suggesting that MutFormer's ability to analyze sequences provides additional information to the protein interpretation problem which evolutionary approaches may not be capable of extracting.

2.4 ProteinGym Evaluation

In the interest of further assessing MutFormer's interpretation ability of protein sequences, we evaluated MutFormer's performance on Deep Mutational Scanning (DMS) data, which corresponds to a prediction of mutation fitness. Mutation evolutionary fitness is a task that is related to pathogenicity, and some methods natively used for predicting evolutionary fitness have been directly ported to pathogenicity prediction. Because evolutionary fitness prediction is not a task that MutFormer is directly optimized for, good performance on DMS data would further indicate MutFormer's general protein comprehensive ability. Using the ProteinGym database provided by Tranception,[4] we compared (external predictions included) MutFormer's performance on this set against all other methods provided by the ProteinGym database. Because some amino acid substitutions present in ProteinGym are not obtainable through SNPs, these amino acid substitutions were not included in our evaluation. After eliminating these examples from all methods' data, we used ProteinGym's evaluation script to run an evaluation of all methods alongside Mutformer on the remaining DMS data. As can be seen from Table S4, MutFormer is outperformed by various methods in the ProteinGym evaluation dataset. We observe that MutFormer, despite being optimized for pathogenicity prediction and not fitness prediction, still shows comparable performance with various current methods in this dataset. The full results of this evaluation are displayed in Table S4.

2.5 Analyzing the weights of MutFormer

The MutFormer model takes advantage of both convolutions and attention, both of which contain useful information about MutFormer's interpretation mechanisms for protein sequences. To better understand these mechanisms, we analyzed MutFormer's model weights for the final best-performing MutFormer model. For our analysis of the convolution layers, two visualizations were generated. In our first visualization, we plotted the average convolution filter weights for each of the four convolution operations. In our second visualization, based on each of the four convolution operations, we plotted the overall percentage consideration given to each input residue id (amino acid identity/special tokens). Both of these visuals are displayed in Figure S5A. Based on visual 2, we see that the overall considerations placed on each residue are very nearly identical across the four different convolutions. Special tokens, as expected, were given very large weight in overall consideration. Interestingly, the "J" token was given a relatively significant weighting, but the "B" token was not as relatively prioritized. Of note, amino acids Serine (S), Cysteine (C), and Valine (V) took more priority relative to other amino acids. Based on visual 1, we can see that first, there are different prioritizations of positions for each of the four convolutions, and additionally, that while specific patterns cannot be directly derived from these filters, it is reasonable to infer that combinations of residue IDs and residue ID patterns are learned by different convolutions.

For our analysis of attention weights, we opted for two case studies from the PDB database: PDB: 1B1C and PDB: 1P4O. The chosen proteins were selected because of their relatively large number of deleterious mutations and complex structure. We ran MutFormer with each protein sequence as input and generated four different visuals: 1) all 8 individual attention maps for each attention layer, 2) a rollout attention map representing the log scale result from taking the dot product over all attention outputs, 3) the distance map between residues within the protein's 3D structure, with each point value given as the negative of the 3D distance between the two residue positions, 4) a deleterious mutation map taken from HGMD data displaying the locations of deleterious mutation sites. All four visuals for each of the two proteins are displayed in Figure S5B. We can see from these visuals that while MutFormer's attention maps for each example do not perfectly correspond to either the 3D distance map or deleterious mutation location map, presumably due to the model's need to consider other relationships simultaneously, there is still notable resemblance present. For instance, in PDB: 1B1C's rollout attention, we find a resemblance with the deleteriousness map, as both have bright bands roughly at positions 12, 48, 74, 120, and 160. We also observe some resemblance between the rollout attention of MutFormer for PDB: 1P4O and the 3D distance map, we roughly observe a bright rectangle from positions 175 to the end of the sequence, a broad band from positions 150 to 200 running the length and width of the rectangle, and a rough rectangle from positions 0 to 100.

Supplemental Figures

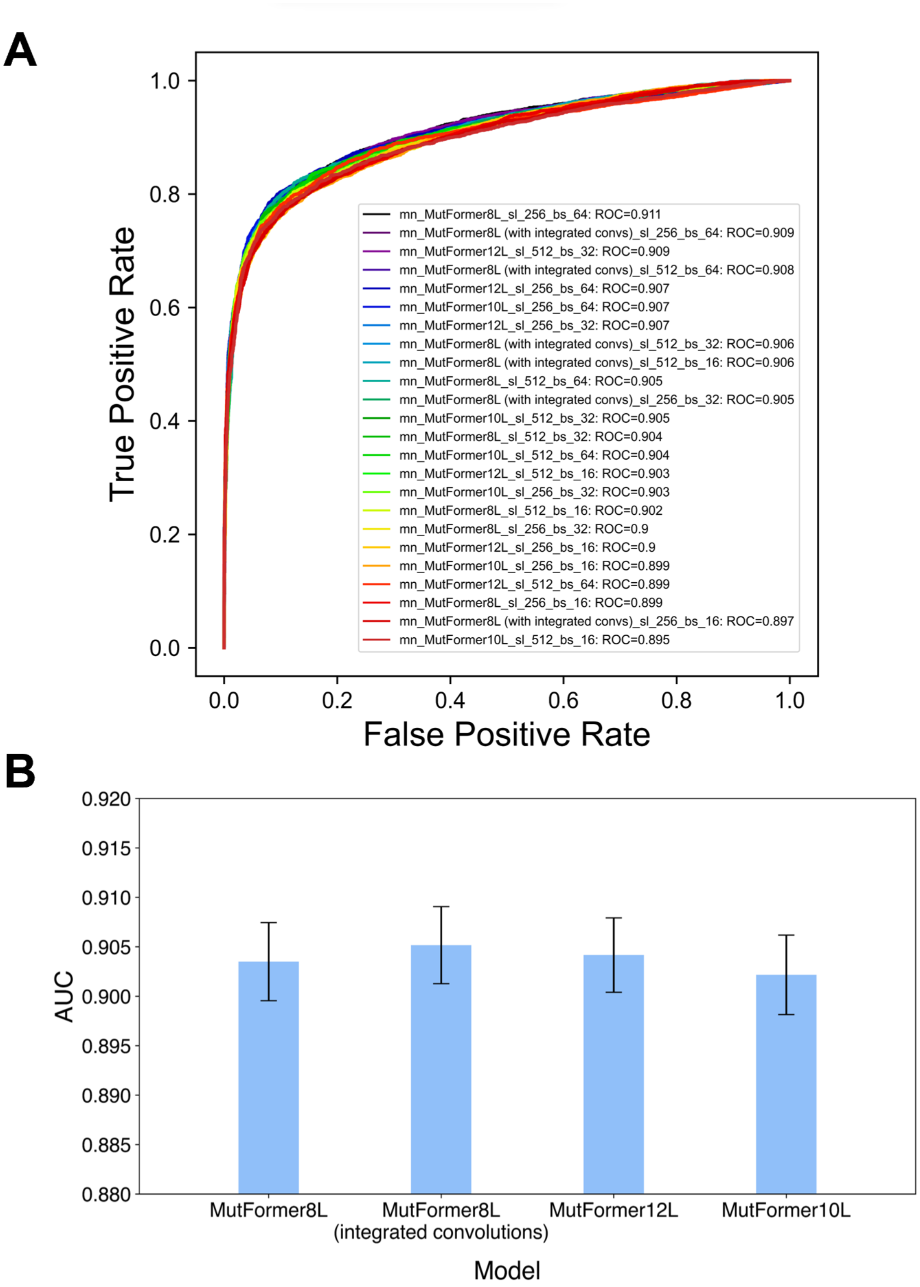

Figure S1

Fine-tuning internal comparison test 2: Performance comarison of MutFormer versus MutFormer *(with integrated convolutions).* **(A)** ROC curves for two different model architectures (class MutFormer and MutFormer (*with integrated convs*)) tested on varying sequence lengths and batch sizes. The labels are in the following format: “mn_[model name]_sl_[max input sequence length]_bs_[batch size]: ROC=[ROCAUC]”. **(B)** Performance comparison of the four different models: MutFormer$_{8L}$*(with integrated convs)*, MutFormer$_{8L}$, MutFormer$_{10L}$, and MutFormer$_{12L}$.

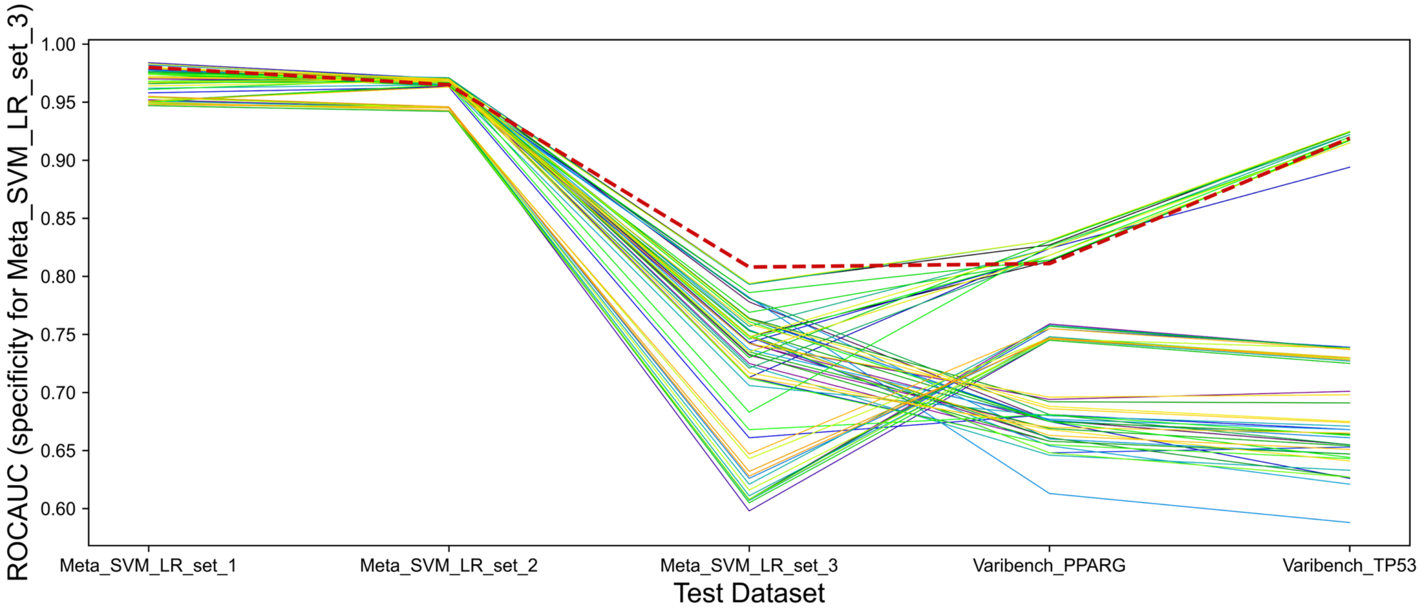

Figure S2

Summary of all testing dataset runs for MutFrmer (with external predictions). All MutFormer test runs for varying levels of “fit” are displayed as solid lines; the chosen best performing overall run, which represented MutFormer in our comparison vs other existing methods, is bolded and dashed. Note that for dataset 3, which contains only negative examples, the y-coordinate in the graph corresponds to specificity instead of ROCAUC.

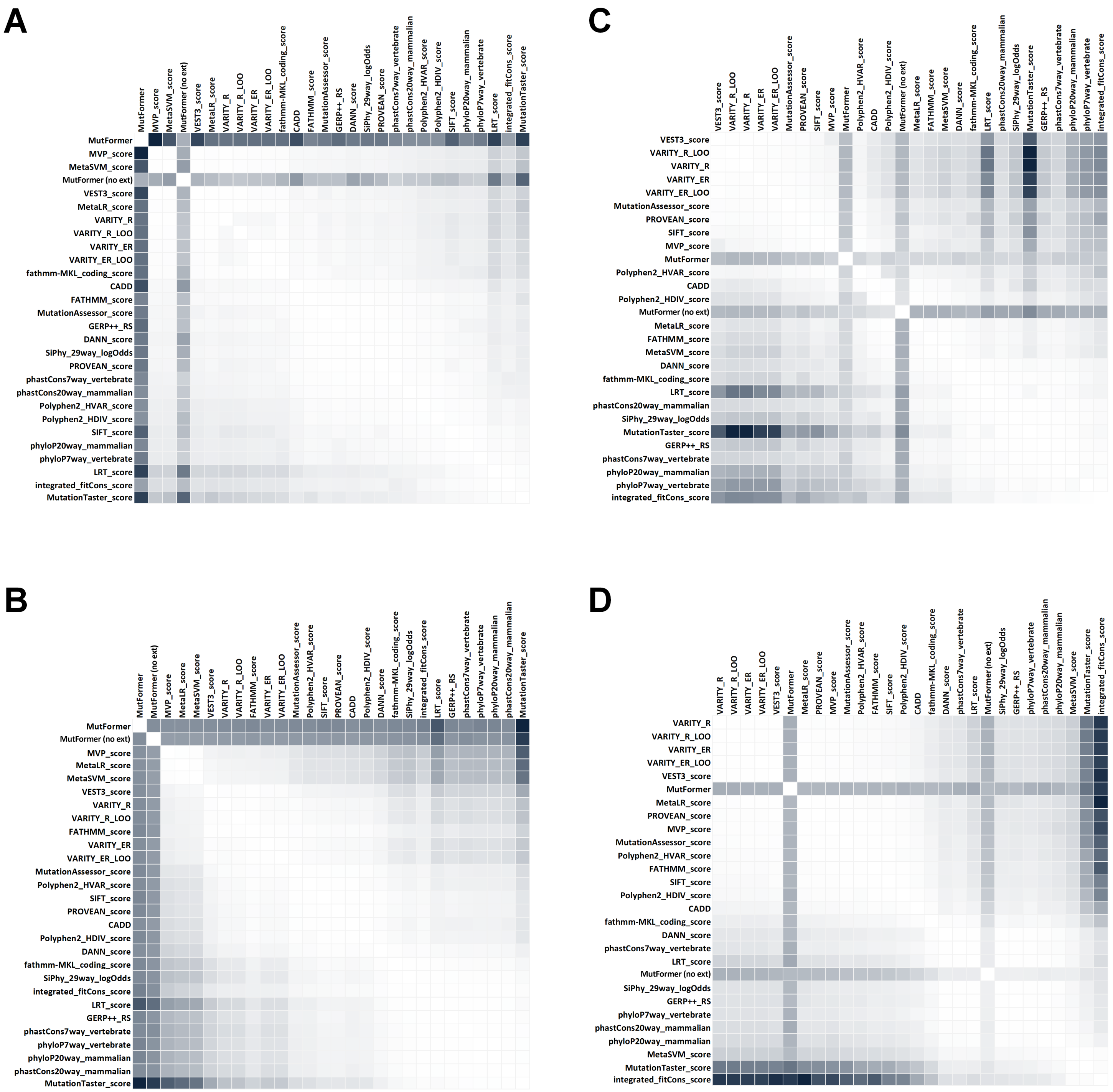

Figure S3

Delong test for MutFormer vs other methods: Delong test reporting statistical probability of equivalence between two ROC curves. Pixel values correspond to log scale probability of equilvalence (darker values indicate lower probability), and the ordering of the methods in the figure is based on their relative performance in each testing dataset. **(A)** Meta_SVM_LR_set_1, **(B)** MetaSVM_LR_set_2, **(C)** Varibench_PPARG, **(D)** Varibench_TP53.

A

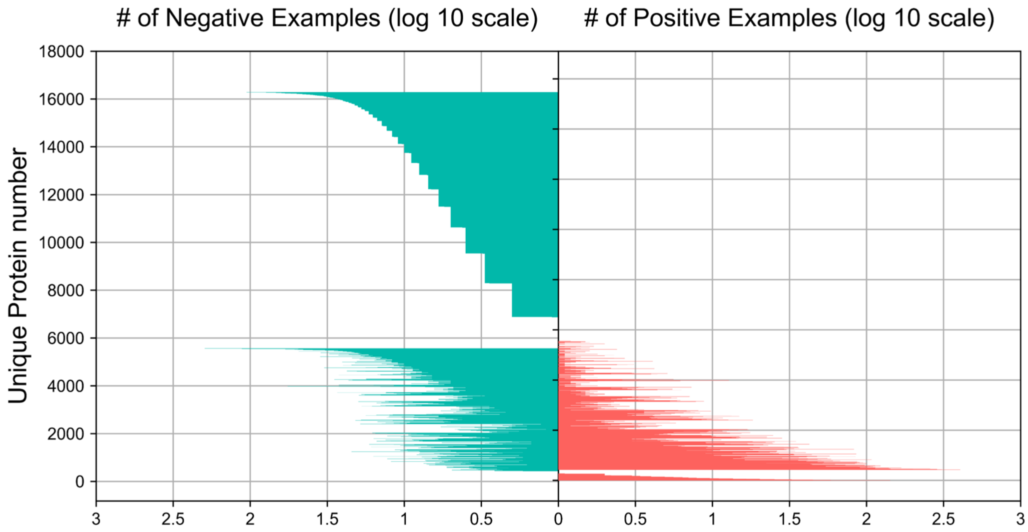

B

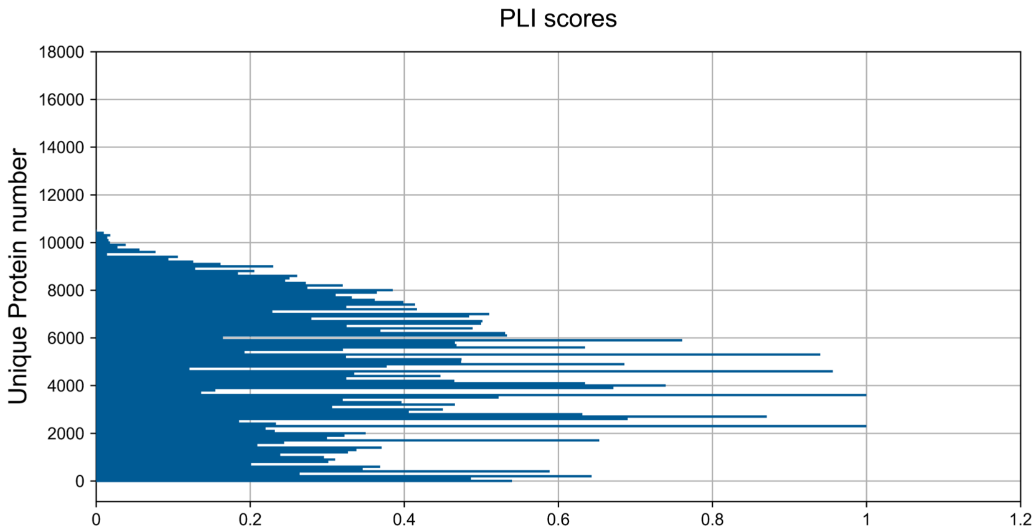

Figure S4

Per Protein Pathogenic and Benign Mutant Counts and Corresponding pLI Scores. Graphical display of the distributions of pathogenic and benign examples included in MutFormer's fine-tuning data at the protein level. Proteins are sorted primarily based on ratio of pathogenic examples to total examples, secondarily by number of pathogenic examples, and tertiarily inversely by the number of benign examples. **(A)** Counts of positive (pathogenic) and negative (benign) mutation examples for each protein ID within all examples present in MutFormer's finetune training data, **(B)** Loss of function intolerance probability (pLI) for each unique protein corresponding to part A based on GnomAD data.

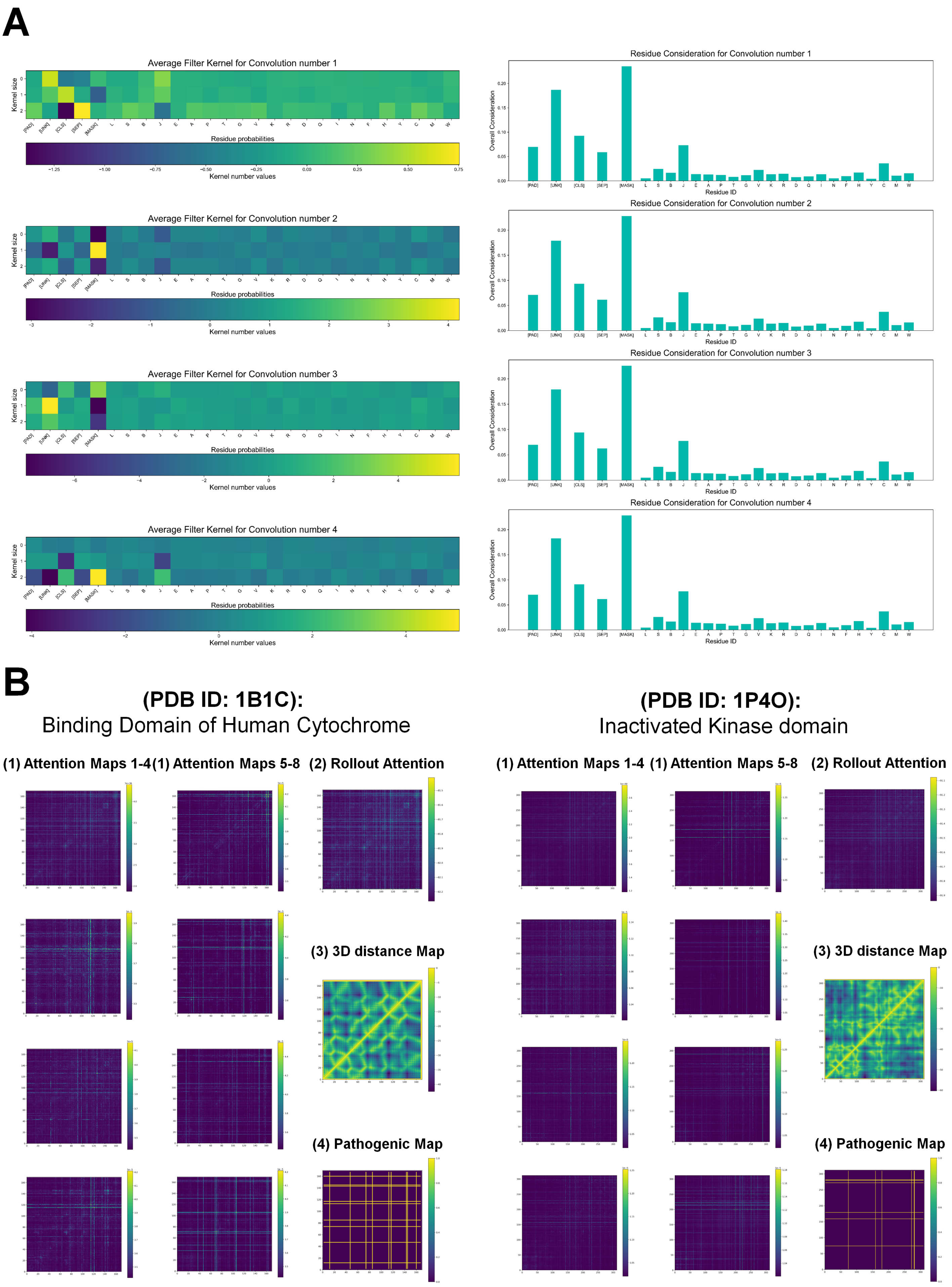
A
Average Filter Kernel for Convolution number 1
Average Filter Kernel for Convolution number 2
Average Filter Kernel for Convolution number 3
Average Filter Kernel for Convolution number 4
Kernel size
Residue probabilities
Kernel number values
Residue Consideration for Convolution number 1
Residue Consideration for Convolution number 2
Residue Consideration for Convolution number 3
Residue Consideration for Convolution number 4
Overall Consideration
Residue ID
[PAD] [UNK] [CLS] [SEP] [MASK] L S B J E A P T G V K R D Q I N F H Y C M W
B
(PDB ID: 1B1C):
Binding Domain of Human Cytochrome
(PDB ID: 1P4O):
Inactivated Kinase domain
(1) Attention Maps 1-4
(1) Attention Maps 5-8
(2) Rollout Attention
(3) 3D distance Map
(4) Pathogenic Map

Figure S5

Model Weights Analysis: Graphical displays of an analysis of Mutformer's attention weights. **(A)** Average convolution filter (left) and overall residue consideration (right) for each of 4 convolutions used by Mutformer, **(B)** Attention weights analysis ((1) attention map for each attention layer, (2) rollout attention: dot producted attention map for all attention layers, (3) 3D distance map for the protein's true 3D structure, and (4) pathogenic map displaying all pathogenic locations found for each protein in the finetuning dataset) for two proteins: PDB: 1B1C (left) and PDB: 1P4O (right).

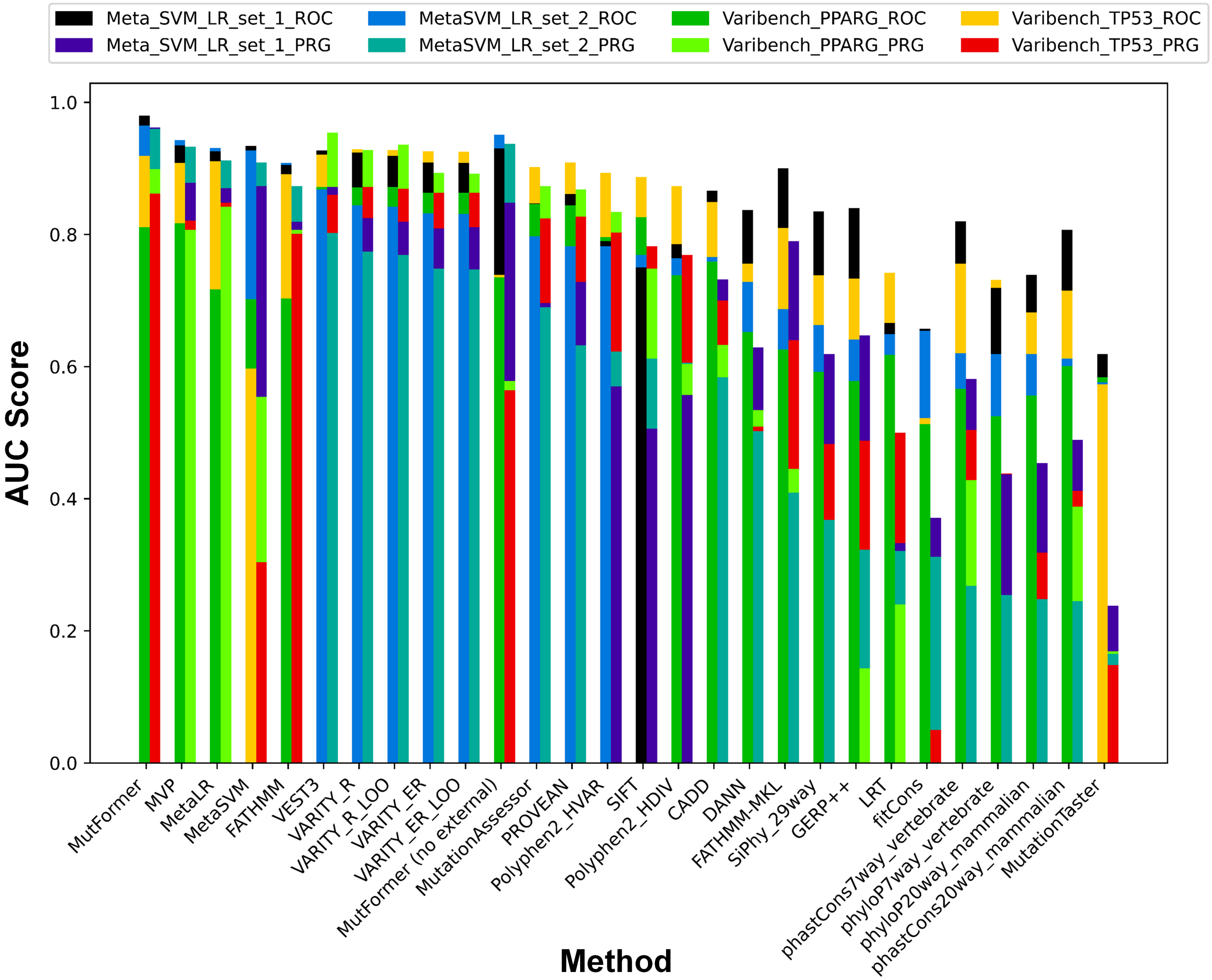

Figure S6

Bar Plot representation of Receiver Operator Characteristic and Precision Recall Gain AUCs for comparison with existing methods. Side by side bar graphs of each method of deleteriousness prediction for their performance as displayed in Figure 4 and 5. For each method, the left bar displays the ROC AUC of that method's performance on datasets 1, 2, 4, and 5. The right bar displays PRG AUC. Note that PRG AUC values that were below 0 were clipped to 0.

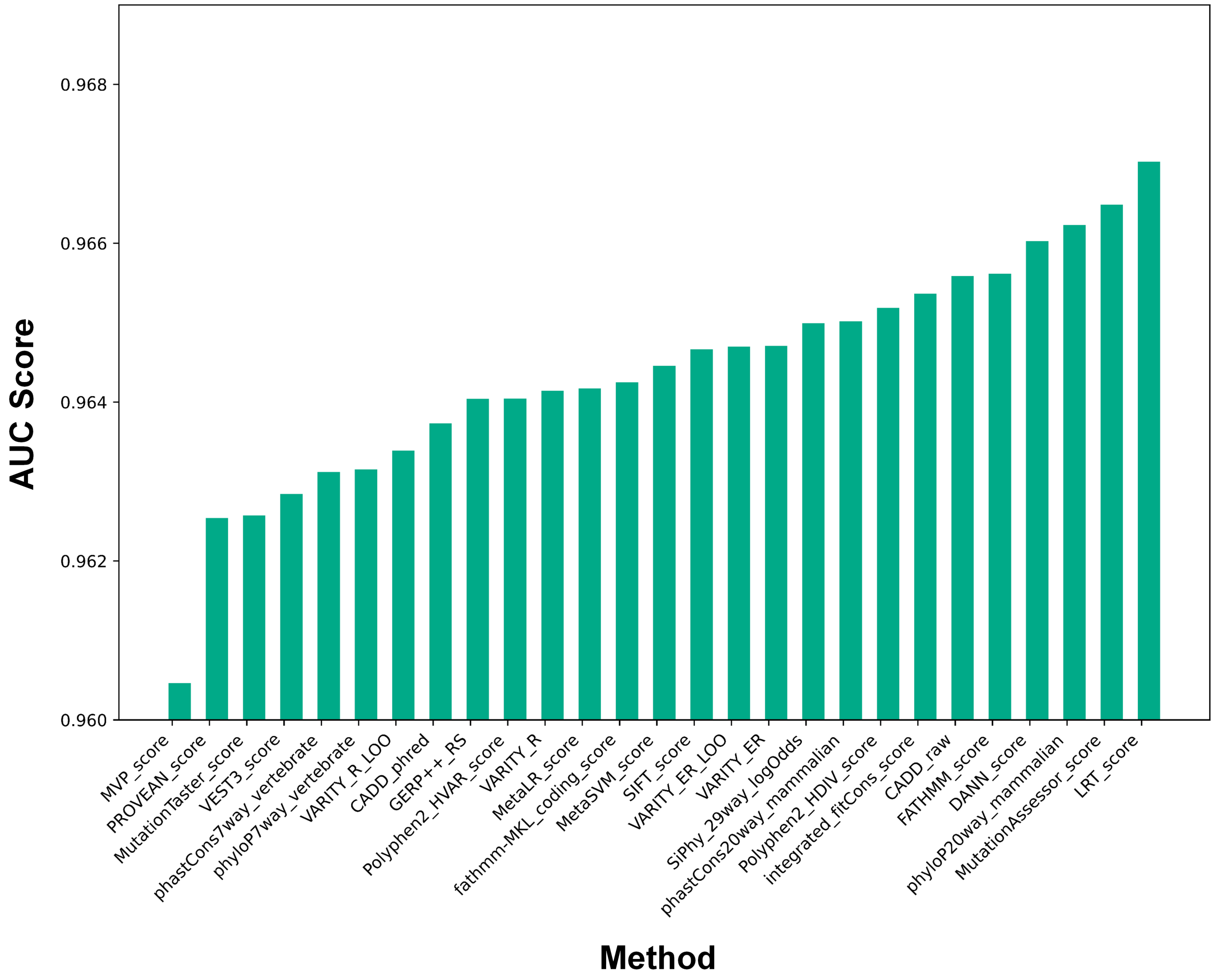

Figure S7

Ablation study results for MutFormer's use of external predictions. ROC AUC performance of MutFormer on our independent validation dataset after removing each external prediction (for each external prediction, all other predictions were kept while all entries for that prediction were set to null). The MutFormer model represented here is MutFormer$_{8L}$ with integrated convolutions, finetuned on a batch size of 32 with 0 freezing layers.

Supplemental Tables

Table S1

Hyperparameters and training description during pretraining for each model.

Model Name	Learning Rate Decay per Step	Batch Size	Steps per Epoch	Total Steps	Training Time
$MutBERT_{8L}$	1.33e-11	64	2300	1.5M	~130 hrs
$MutBERT_{10L}$	1.33e-11	64	2300	1.5M	~160 hrs
$MutFormer_{8L}$	1.33e-11	64	2300	1.5M	~150 hrs
$MutFormer_{10L}$	1.33e-11	64	2300	1.5M	~175 hrs
$MutFormer_{12L}$	1.00e-11	32	4600	2.0M	~275 hrs
$MutFormer_{8L}$ *(with integrated convs)*	1.33e-11	64	2300	1.5M	~150 hrs

Additional hyperparameters constant for all models:

- Initial Learning Rate: 2e-5
- Weight Decay (For Adam Optimizer): 0.01
- Gradient Clip amount (During Optimization): 1.0

Table S2

Loss and accuracy on the pretraining task (masked residue prediction).

Model Name	Training split		Test split	
	Loss	Accuracy	Loss	Accuracy
$MutBERT_{8L}$	1.4641	0.5538	2.0297	0.4021
$MutBERT_{10L}$	1.1360	0.6504	1.7248	0.4863
$MutFormer_{8L}$	0.9872	0.6984	1.2274	0.6212
$MutFormer_{10L}$	0.8560	0.7384	1.0961	0.6631
$MutFormer_{12L}$	0.8338	0.7460	1.0727	0.6730
$MutFormer_{8L}$ *(with integrated convs)*	0.8305	0.7475	1.0590	0.6930

Table S3

The numbers of SNVs in the test sets that were missing from each method.

Method	Reference	Number of missing SNVs					
		Set 1	Set 2	Set 3	Set 4	Set 5	Set 6
MutFormer	-	0	0	0	0	0	0
SIFT	[5]	16	2	203	72	102	104
PolyPhen2-HDIV	[6]	5	0	100	22	21	15
PolyPhen2-HVAR	[6]	5	0	100	22	21	15
LRT	[7]	130	10	701	22	501	256
MutationTaster	[8]	8	0	65	25	14	14
MutationAssessor	[9]	27	2	228	23	64	69
FATHMM	[10]	142	2	484	50	186	207
PROVEAN	[11]	12	1	160	67	46	51
VEST3	[12]	1	0	74	22	13	13
CADD	[13,14]	0	0	46	22	0	0
DANN	[15]	0	0	46	22	0	0
FATHMM-MKL	[16]	0	0	46	22	0	0
MetaSVM	[17]	1	0	46	22	3	3
MetaLR	[17]	1	0	46	22	3	3
fitCons	[18]	87	3	1195	159	433	433
GERP++	[19]	0	0	50	22	7	7
PhyloP-7way-vertebrate	[20]	0	0	47	22	1	1
PhyloP-20way-mammalian	[20]	0	0	47	22	0	0
PhastCons-7way-vertebrate	[21]	0	0	47	22	1	1
PhastCons-20way-mammalian	[21]	0	0	47	22	0	0
SiPhy-29way (log odds)	[22]	2	0	68	22	12	9
VARITY_ER	[23]	95	6	516	77	428	200
VARITY_ER_LOO	[23]	95	6	516	77	428	200
VARITY_R	[23]	95	6	516	77	428	200
VARITY_R_LOO	[23]	95	6	516	77	428	200
MVP	[24]	0	4	969	6	228	171

Note: the total numbers of SNVs in the test sets are outlined in Table 2.

Table S4

The ROC (Receiver Operator Characteristic) and RPG (Precision- Recall- Gain) score of various methods on the testing dataset. This table is provided as a separate CSV file due to the presence of many columns.

Table S5

ProteinGym Evaluation results between MutFormer and other methods included in ProteinGym.

Method	ROC AUC Score	Matthews Correlation Coefficient	Spearman Rank Correlation
Ensemble Tranception & EVE	0.756	0.35	0.435
ESM-1v (ensemble)	0.751	0.341	0.418
Tranception M	0.745	0.339	0.415
Tranception L	0.744	0.335	0.413
EVE (ensemble)	0.738	0.334	0.403
Tranception S	0.735	0.329	0.398
DeepSequence (ensemble)	0.735	0.326	0.396
EVE (single)	0.735	0.324	0.395
Progen2 (ensemble)	0.733	0.322	0.394
ESM-1v (single)	0.732	0.322	0.39
DeepSequence (single)	0.732	0.321	0.39
MSA Transformer (ensemble)	0.729	0.317	0.39
Progen2 Base	0.729	0.316	0.379
Wavenet	0.728	0.315	0.379
EVmutation	0.725	0.313	0.378
MSA Transformer (single)	0.724	0.311	0.376
Progen2 M	0.724	0.306	0.376
Mutformer	0.723	0.305	0.376
Progen2 L	0.723	0.304	0.375
RITA (ensemble)	0.723	0.301	0.373
Progen2 S	0.717	0.3	0.367
RITA L	0.717	0.298	0.362
Tranception L no retrieval	0.713	0.296	0.359
Site-Independent	0.712	0.294	0.358
RITA M	0.711	0.288	0.354
RITA XL	0.709	0.284	0.348
Progen2 XL	0.702	0.271	0.338
RITA S	0.682	0.245	0.306

References

1. Karczewski, K.J., Francioli, L.C., Tiao, G., et al. (2020). The mutational constraint spectrum quantified from variation in 141,456 humans. Nature ***581***, 434-443.
2. Stenson, P.D., Mort, M., Ball, E.V., et al. (2020). The Human Gene Mutation Database (HGMD((R))): optimizing its use in a clinical diagnostic or research setting. Hum Genet ***139***, 1197-1207.
3. Frazer, J., Notin, P., Dias, M., et al. (2021). Disease variant prediction with deep generative models of evolutionary data. Nature ***599***, 91-95.
4. Notin, P., Dias, M., Frazer, J., et al. (2022). Tranception: protein fitness prediction with autoregressive transformers and inference-time retrieval. International Conference on Machine Learning. PMLR.
5. Ng, P.C., and Henikoff, S. (2003). SIFT: Predicting amino acid changes that affect protein function. Nucleic Acids Res ***31***, 3812-3814.
6. Adzhubei, I.A., Schmidt, S., Peshkin, L., et al. (2010). A method and server for predicting damaging missense mutations. Nat Methods ***7***, 248-249.
7. Chun, S., and Fay, J.C. (2009). Identification of deleterious mutations within three human genomes. Genome Res ***19***, 1553-1561.

8. Schwarz, J.M., Cooper, D.N., Schuelke, M., and Seelow, D. (2014). MutationTaster2: mutation prediction for the deep-sequencing age. Nat Methods ***11***, 361-362.
9. Reva, B., Antipin, Y., and Sander, C. (2011). Predicting the functional impact of protein mutations: application to cancer genomics. Nucleic Acids Res ***39***, e118.
10. Shihab, H.A., Gough, J., Cooper, D.N., et al. (2013). Predicting the functional, molecular, and phenotypic consequences of amino acid substitutions using hidden Markov models. Hum Mutat ***34***, 57-65.
11. Choi, Y., Sims, G.E., Murphy, S., et al. (2012). Predicting the functional effect of amino acid substitutions and indels. PLoS One ***7***, e46688.
12. Carter, H., Douville, C., Stenson, P.D., et al. (2013). Identifying Mendelian disease genes with the variant effect scoring tool. BMC Genomics ***14 Suppl 3***, S3.
13. Rentzsch, P., Witten, D., Cooper, G.M., et al. (2019). CADD: predicting the deleteriousness of variants throughout the human genome. Nucleic Acids Res ***47***, D886-D894.
14. Kircher, M., Witten, D.M., Jain, P., et al. (2014). A general framework for estimating the relative pathogenicity of human genetic variants. Nat Genet ***46***, 310-315.
15. Quang, D., Chen, Y., and Xie, X. (2015). DANN: a deep learning approach for annotating the pathogenicity of genetic variants. Bioinformatics ***31***, 761-763.
16. Shihab, H.A., Rogers, M.F., Gough, J., et al. (2015). An integrative approach to predicting the functional effects of non-coding and coding sequence variation. Bioinformatics ***31***, 1536-1543.
17. Dong, C., Wei, P., Jian, X., et al. (2015). Comparison and integration of deleteriousness prediction methods for nonsynonymous SNVs in whole exome sequencing studies. Hum Mol Genet ***24***, 2125-2137.
18. Gulko, B., Hubisz, M.J., Gronau, I., and Siepel, A. (2015). A method for calculating probabilities of fitness consequences for point mutations across the human genome. Nat Genet ***47***, 276-283.
19. Davydov, E.V., Goode, D.L., Sirota, M., et al. (2010). Identifying a high fraction of the human genome to be under selective constraint using GERP++. PLoS Comput Biol ***6***, e1001025.
20. Pollard, K.S., Hubisz, M.J., Rosenbloom, K.R., and Siepel, A. (2010). Detection of nonneutral substitution rates on mammalian phylogenies. Genome Res ***20***, 110-121.
21. Siepel, A., Bejerano, G., Pedersen, J.S., et al. (2005). Evolutionarily conserved elements in vertebrate, insect, worm, and yeast genomes. Genome Res ***15***, 1034-1050.
22. Garber, M., Guttman, M., Clamp, M., et al. (2009). Identifying novel constrained elements by exploiting biased substitution patterns. Bioinformatics ***25***, i54-62.
23. Wu, Y., Li, R., Sun, S., et al. (2021). Improved pathogenicity prediction for rare human missense variants. Am J Hum Genet.
24. Qi, H., Zhang, H., Zhao, Y., et al. (2021). MVP predicts the pathogenicity of missense variants by deep learning. Nat Commun ***12***, 510.
