# Supplementary material for: Deciphering “the language of nature”: A transformer-based language model for deleterious mutations in proteins
Source: Innovation (Camb). 2023 Jul 27;4(5):100487. doi: 10.1016/j.xinn.2023.100487 (PMC10448337; doi:10.1016/j.xinn.2023.100487)
Supplement: Document S2. Article plus supplemental information [file mmc3.pdf]

www.the-innovation.org

Deciphering “the language of nature”: A transformer-based language model for deleterious mutations in proteins

Theodore T. Jiang,[1,2,3] Li Fang,[1,4,*] and Kai Wang[1,5,*]
*Correspondence: fangli9@mail.sysu.edu.cn (L.F.); wangk@chop.edu (K.W.)

Received: February 9, 2023; Accepted: July 25, 2023; Published Online: July 27, 2023; https://doi.org/10.1016/j.xinn.2023.100487

© 2023 The Authors. This is an open access article under the CC BY license (http://creativecommons.org/licenses/by/4.0/).

GRAPHICAL ABSTRACT

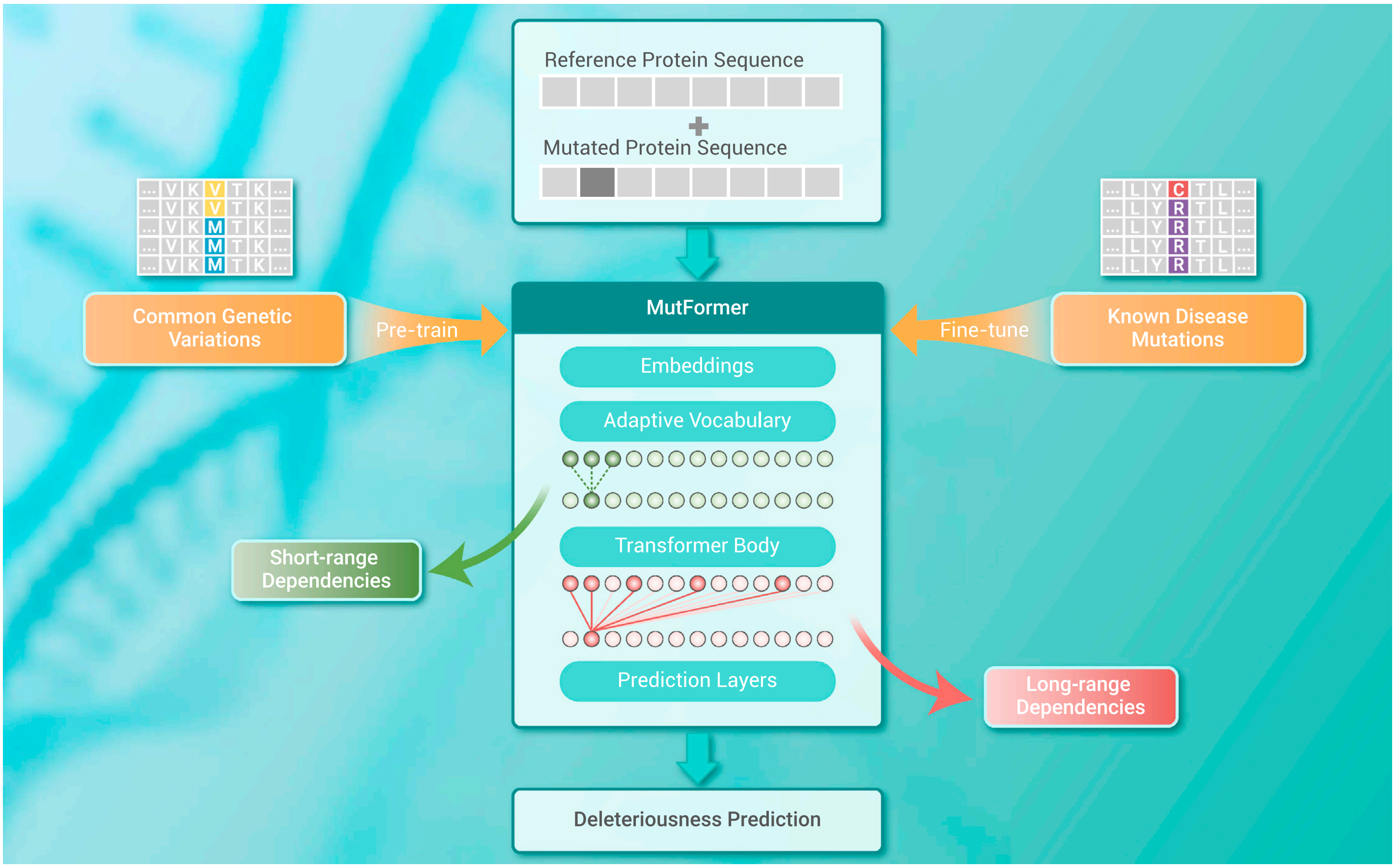

PUBLIC SUMMARY

- Prediction of deleterious mutations using a model inspired from natural language processing.
- A transformer-based model to learn long- and short-range dependencies between amino acids.
- Learning from both common genetic variations and known disease mutations.
- Complements existing predictions or empirically generated functional scores.

Deciphering "the language of nature": A transformer-based language model for deleterious mutations in proteins

Theodore T. Jiang,[1,2,3] **Li Fang,**[1,4,*] **and Kai Wang**[1,5,*]

[1]Raymond G. Perelman Center for Cellular and Molecular Therapeutics, Children's Hospital of Philadelphia, Philadelphia, PA 19104, USA

[2]Palisades Charter High School, Pacific Palisades, CA 90272, USA

[3]Massachusetts Institute of Technology, Cambridge, MA 02139, USA

[4]Department of Genetics and Biomedical Informatics, Zhongshan School of Medicine, Sun Yat-sen University, Guangzhou 510080, China

[5]Department of Pathology and Laboratory Medicine, Perelman School of Medicine, University of Pennsylvania, Philadelphia, PA 19104, USA

*Correspondence: fangli9@mail.sysu.edu.cn (L.F.); wangk@chop.edu (K.W.)

Received: February 9, 2023; Accepted: July 25, 2023; Published Online: July 27, 2023; https://doi.org/10.1016/j.xinn.2023.100487

© 2023 The Authors. This is an open access article under the CC BY license (http://creativecommons.org/licenses/by/4.0/).

Citation: Jiang T.T., Fang L., and Wang K. (2023). Deciphering "the language of nature": A transformer-based language model for deleterious mutations in proteins. The Innovation **4(5)**, 100487.

Various machine-learning models, including deep neural network models, have already been developed to predict deleteriousness of missense (non-synonymous) mutations. Potential improvements to the current state of the art, however, may still benefit from a fresh look at the biological problem using more sophisticated self-adaptive machine-learning approaches. Recent advances in the field of natural language processing show that transformer models—a type of deep neural network—to be particularly powerful at modeling sequence information with context dependence. In this study, we introduce MutFormer, a transformer-based model for the prediction of deleterious missense mutations, which uses reference and mutated protein sequences from the human genome as the primary features. MutFormer takes advantage of a combination of self-attention layers and convolutional layers to learn both long-range and short-range dependencies between amino acid mutations in a protein sequence. We first pre-trained MutFormer on reference protein sequences and mutated protein sequences resulting from common genetic variants observed in human populations. We next examined different fine-tuning methods to successfully apply the model to deleteriousness prediction of missense mutations. Finally, we evaluated MutFormer's performance on multiple testing datasets. We found that MutFormer showed similar or improved performance over a variety of existing tools, including those that used conventional machine-learning approaches. In conclusion, MutFormer considers sequence features that are not explored in previous studies and can complement existing computational predictions or empirically generated functional scores to improve our understanding of disease variants.

INTRODUCTION

Whole-exome and whole-genome sequencing technologies are powerful tools for the detection of genetic mutations. A typical human genome has 4.1 million to 5.0 million variants when compared with the reference genome sequence,[1] while the average exome captures genomic regions that account for 1%–2% of the human genome. Therefore, distinguishing or prioritizing a small number of disease-related variants from such a large number of background variants becomes a key challenge in understanding genome and exome sequencing data. In particular, the interpretation of non-synonymous single nucleotide variants (SNVs) is of major interest, because missense mutations in proteins account for more than one-half of the current known variants responsible for human-inherited disorders, especially Mendelian diseases, where the mutations have high penetrance.[2] Unlike frameshift indels or splicing mutations in canonical splice sites that have a high likelihood to alter protein function, missense mutations change only a single amino acid, so most of them may not have significant impacts on protein function. To this end, population-specific allele frequencies, such as those inferred from the ExAC[3,4] and gnomAD[5] databases, can be useful to filter out common missense variants that are likely to be neutral, and mutation databases such as ClinVar[6–8] and the Human Gene Mutation Database (HGMD)[2] can be valuable resources to find previously reported mutations that may be deleterious. Still, a large number of missense variants from exome sequencing are not yet documented; therefore, the functional interpretation of such variants remains a crucial task.

Numerous computational tools have been developed to predict the deleteriousness or pathogenicity of missense mutations[9–16]; however, as shown by multiple recent publications, the accuracy of predictive algorithms still has room for improvement. Databases such as dbNSFP[9–11] have now documented these whole-exome prediction scores for different prediction algorithms in an effort to facilitate the development of improved functional assessment algorithms. However, depending on the evaluation datasets that were used, most algorithms for missense variant prediction are 65%–80% accurate when examining known disease variants, and only approximately 43.4% of pairwise prediction correlations between different predictive algorithms are greater than 0.5.[9] Many conflicting predictions can be made between different algorithms, which motivated the development of several ensemble-based scoring systems that combine multiple prediction algorithms, such as MetaSVM,[17] REVEL,[18] and CADD.[19] In fact, predictions combined from different algorithms are considered as a single piece of evidence according to the American College of Medical Genetics and Genomics-Association for Molecular Pathology 2015 guidelines.[20] In addition, most existing computational algorithms are based on similar or related information (e.g., evolutionary conservation scores, mutation tolerance scores); potential improvements to the current state of the art could benefit from a fresh look at the biological problem using more sophisticated self-adaptive machine-learning approaches that examine additional types of information.

In other previously published prediction algorithms, deep learning-based sequence-focused models have been demonstrated as effective in modeling variant function. These existing methods primarily used convolutional neural networks (CNNs) to model sequences.[21–23] However, recently, advances in deep learning have shown transformer models to be particularly powerful for modeling sequential data. Transformer models, such as the Bidirectional Encoder Representations from Transformers (BERT),[24,25] rely on its central mechanism, self-attention. The use of self-attention allows the transformer model to achieve an unprecedented ability to model relationships between tokens in a sequence, which is crucial in the comprehension of linear sequences. In the past three years, transformers have achieved state-of-the-art performances on a broad range of natural language processing (NLP) tasks,[24,26–28] and transformers are competitive with more traditional CNN-based models on image recognition tasks.[29] As of late, transformers have also been successfully applied for modeling protein structure in Alphafold2,[30] and in works such as Enformer,[31] which used transformers for DNA interpretation. Part of the reasons for the successes of transformers may be caused by their increased ability to handle subtle context dependency through a multi-head attention mechanism, and the ability to compute attentions in parallel to greatly speed up computation over typical recurrent neural network-based algorithms.

In biological contexts, each amino acid in a given protein sequence exerts its function in a context-dependent manner, including both local dependency (such as forming a short signal peptide that was recognized by cellular machinery) and long-distance dependency (such as being close to another amino acid in three-dimensional structure to form a binding site for ligands). Therefore, we hypothesize that transformer models would be capable of more effective modeling of protein sequences, somewhat similar to how transformers have transformed the field of NLP and language translation over the past few years.

In this study, we propose MutFormer, a transformer-based model, to assess deleteriousness of missense mutations. MutFormer is an adaption of the BERT architecture[24] to protein contexts, with appropriate modifications to

incorporate protein-specific characteristics. MutFormer can analyze protein sequences directly, with or without any homology information or additional data. Our experiments show that MutFormer is capable of matching or outperforming current methods in the deleteriousness prediction of missense variants.

MutFormer is based on the BERT architecture.[24] A central component of the classical BERT model is its bidirectional self-attention. This mechanism uses a two-dimensional matrix to model the context between all positions in a given sequence, enabling efficient learning of long-range dependencies between residues. In contrast, convolution is another mechanism capable of learning dependencies, which is better suited for short-range dependencies: convolutions are more capable of prioritizing localized patterns via filters, while the repeated application of convolution filters, which are required for the relating of farther residues in a sequence, often weakens long-range dependencies. MutFormer takes advantage of both self-attention layers and convolutional layers to effectively learn both long-range and short-range dependencies.

In language modeling tasks, words or sub-words are short-range features of the sequence. The original BERT uses a fixed WordPiece vocabulary, which contains common words or sub-words in the training corpus.[32] This vocabulary cannot be tuned during the pre-training and fine-tuning process; therefore, a spelling error may introduce an out-of-vocabulary word that will hinder the model's interpretation ability of a given sequence. In protein sequences, "words" correspond with key subsequences or patterns of amino acids. These words can often be changed because of mutations, and furthermore, are unknown. Recent studies showed that vocabulary-free models (e.g., byte-level models) are more robust to noise and perform better on tasks that are sensitive to spelling.[33] Therefore, instead of using a fixed vocabulary, convolutional layers placed in between the embedding layers and the transformer body are used by MutFormer. MutFormer uses these convolutions to learn its own vocabulary over the course of the training process, incorporating nonlinear patterns via the convolution filters. The weights of the convolutional layers are tuned during both the pre-training and fine-tuning processes.

MATERIALS AND METHODS

Pre-processing of input sequence

The input of the MutFormer model is an amino acid sequence that can be either a single protein (with a missense mutation) or the concatenation of a pair of proteins (a mutated protein and its corresponding reference protein). Each amino acid is considered as a token. The maximum input length of MutFormer was set to 1,024, where protein sequences longer than 1,024 need to be cut into segments (see Supplemental Methods 1.1 for details). In the pre-processing step, some special tokens were added to the sequence (Figure 1A). As the input sequence may be a cropped sequence, we add a "B" token to the real start position of the protein and a "J" token to the real end of the protein so that a cropped start/end (without B or J) can be distinguished from a real start/end (with B or J). B and J letters were chosen because they are not included in the current amino acid code table. In the original BERT model, the first token of every sequence is always a [CLS] token and the final hidden state corresponding with this token is used as the aggregate sequence representation for classification tasks; a [SEP] token is used to separate different sentences and is also placed at the very end of the input.[24] We followed this practice in MutFormer (Figure 1A).

The MutFormer model

The MutFormer architecture was implemented on top of the classic BERT architecture. The MutFormer model consists of three primary parts: embeddings, convolutions, and the transformer body (Figure 1B).

The embedding layers create positional, label, and token embeddings for the input sequence. We denote the input sequence length as S, and hidden embedding size as H. Positional embeddings are calculated based on a learnable parameter matrix (size: $S \times H$). Label embeddings are one-hot encodings that indicate whether the amino acid belongs to the reference or mutated sequence. Label embeddings are only used for paired sequence input and are not used if the input is a single protein. Token embeddings map each amino acid into a high dimensional space (dimension = H). Token embeddings are real numbers and are learnable parameters (size: $S \times H$). The final embedding output is the sum of these three embedding representations with layer normalization applied after the sum.

For the convolutions of MutFormer, two different integrations into the model were tested: (1) four convolution layers, where the convolution outputs are the only input to the following transformer body, and (2) an integrated approach with skip connections in which the original embedding outputs and convolution outputs are combined and fed into the transformer body (the input of the transformer body is a sum of the embedding output and the result of applying two or four convolutions). Each convolution used a kernel size of 3, and no pooling or concatenation was applied (only the convolution operation itself was necessary to take advantage of convolutions' pattern recognition ability).

The transformer body of MutFormer is taken from the original BERT model, along with its self-attention modules. The time and space complexity of self-attention is quadratic in the length of input.[34] Because of the constraint of computational resources, a maximum input sequence length needs to be set. Sequences exceeding the maximum sequence length are trimmed. Regardless of the trim position, the position embedding corresponding to first position was always assigned to the first residue in the trimmed sequence but not the full sequence. For this reason, the position embeddings represent more of a relative position rather than true position within the full protein sequence.

Pre-training of MutFormer on human protein sequences

MutFormer was pre-trained on a database obtained by combining human reference protein sequences (all isoforms) and protein sequences caused by non-synonymous SNVs with more than 1% population frequency in the gnomAD database.[5] For a full description of the pre-training data preparation, see Supplemental Methods 1.1.

The original BERT model uses a self-supervised pre-training objective of recovering an original sequence from corrupted (masked) input, from which high-dimensional representations of the sequence are learned. Similar to BERT, the pre-training objective of MutFormer was to predict corrupted amino acid residues from altered sequences. For the corrupted/masked residue prediction task, for each sequence, a number of residues were randomly selected for corruption. The ones that were selected were corrupted by either (1) replacing them with a [MASK] token or (2) another random amino acid. This was done to encourage the learning of context not only around explicitly masked residues, but on the entire sequence. To ensure enough context was present for the model, a maximum number of 20 amino acids were masked per sequence. This maximum number was chosen through testing; we tested training using a fixed masking percentage of 15%; however, this resulted in non-convergence during pre-training. For this reason, the 20-residue cap was used alongside the 15% maximum, whichever one was lower, to determine the number of residues masked. To facilitate the learning of more dependency knowledge and minimize overfitting, we used dynamic masking throughout the duration of the training: the corrupted residues were changed randomly for each epoch of data trained on. Note that, for MutFormer, the "next sentence prediction" objective used by the original BERT was removed, because protein sequences in aggregate, unlike their natural language counterpart, do not form 'paragraphs' with logical connections between sentences. The pre-training was performed on a single cloud machine instance with one tensor processing unit (TPU) hardware accelerator (TPU v2-8) on the Google Cloud Platform. Depending on the model, pre-training took approximately 100–200 h.

We pre-trained MutFormer with three different model sizes (Table 1), as well as a MutFormer model with integrated convolutions. For comparison purposes, we also pre-trained two models without convolutions, which were designated MutBERT to indicate the use of the original BERT architecture (Table 1). The hyperparameters used for pre-training as well as training time, for all models, are displayed in Table S1, and the loss and accuracy for the pre-training task are listed in Table S2.

Fine-tuning MutFormer for the prediction of deleterious mutations

MutFormer was fine-tuned on a dataset built from 84K manually annotated pathogenic missense SNVs from the HGMD (version 2016)[2] and SNPs from the gnomAD database[5] with an allele frequency of more than 0.1%. We generated a training and independent validation set from this data. Mutated protein sequences were generated using ANNOVAR,[35] with each sequence containing exactly one mutation. The fine-tuning was performed on a single cloud machine instance with one TPU hardware accelerator (TPU v2-8) on the Google Cloud Platform. While the specific training time varies from model to model, training time corresponded with approximately 200 steps per minute, making the general time range for fine-tuning approximately 1 h. To obtain the best possible results in deleteriousness prediction, we tested three different fine-tuning methods (described below).

Per residue classification. The single mutated protein sequence is used as input. The model is tasked with classifying each amino acid in the protein sequence as benign or deleterious. Amino acids that are identical to the reference sequence are labeled as benign, and the mutated residue is labeled as benign if the overall mutation is benign, or deleterious if the overall mutation is deleterious, depending on the true classification of the sequence (loss and metrics for classification are calculated on only the mutation site). This corresponds to the token classification task or named entity recognition task in NLP (Figure 2A).

Single sequence classification. Same as per residue classification, the input is a single mutated protein sequence. The model is tasked with classifying the entire sequence as deleterious or benign (via the [CLS] token). This similar to the sentence classification task (e.g., sentiment analysis) in NLP (Figure 2B).

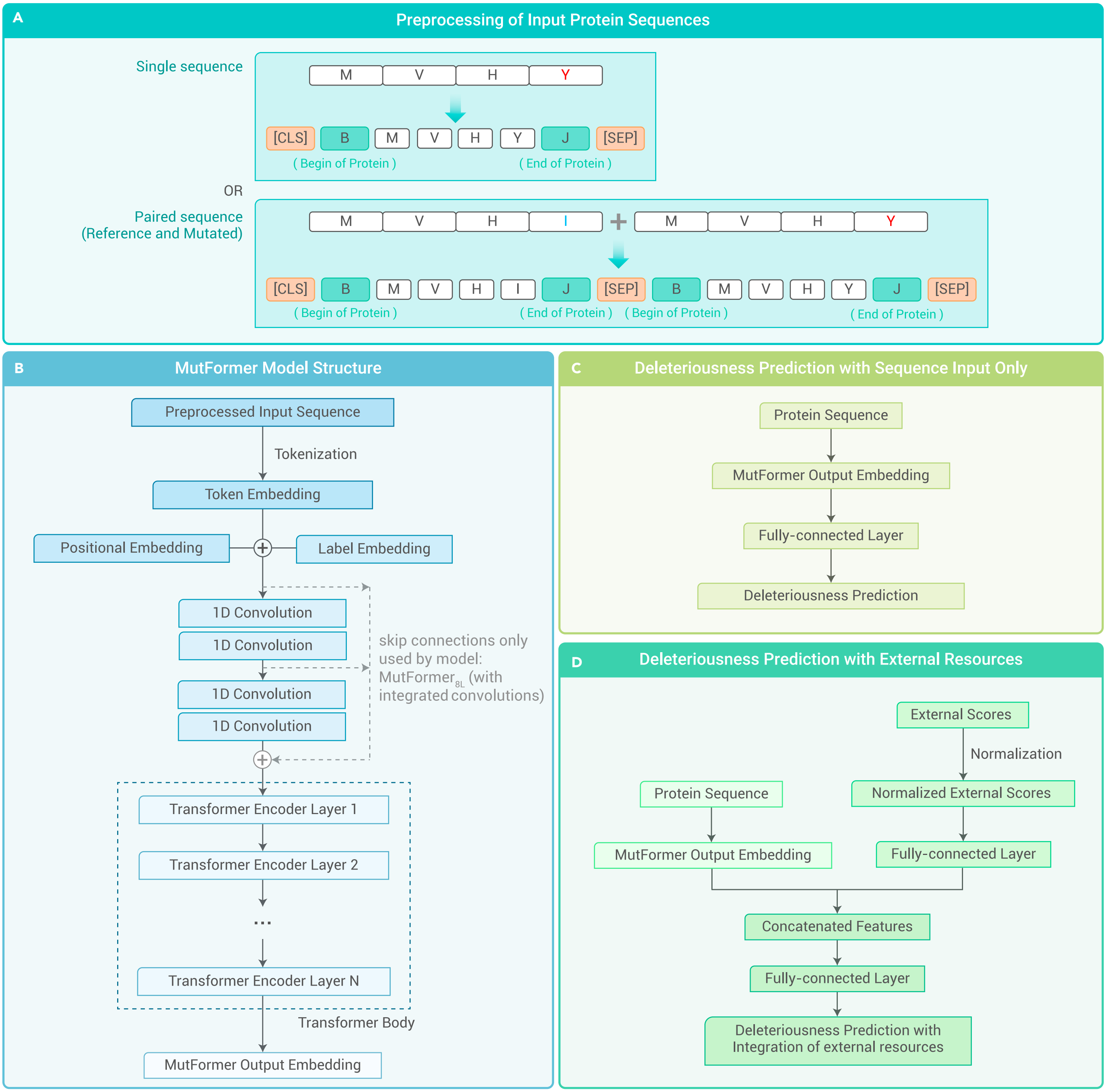

Figure 1. The MutFormer model architecture (A) Pre-processing procedure of input. The input may be a single protein (with a missense mutation) or a pair of protein sequences (a reference protein and a mutated protein). In the input sequences, the red color indicates the mutated amino acid and the blue color indicates the original amino acid in the reference protein sequence. For the single-sequence case, "B" and "J" tokens are attached to the true start and end of the sequence, respectively. The sequence is then cropped to the maximum sequence length. A [CLS] token and a [SEP] token are then added to the start and end of the whole sequence, respectively. For the paired-sequence case, the B and J tokens are added as normal to both sequences, except now a [CLS] token is placed at the beginning of the first sequence; a [SEP] token is placed between the two; and another [SEP] token is placed at the very end. (B) MutFormer model structure. A system of positional, label, and token embeddings is used to first vectorize the input tokens. Two convolutional operations (four convolution layers (kernel size 3, stride 1, filters 768)) are used to process the embedding representation (for the integrated convolutions model, skip connections are used before both convolution operations). A bidirectional transformer body with self-attention applies a sequence of attention layers to the resulting embedding representation to obtain the output embeddings (MutFormer/MutBERT$_{NL}$ indicates N layers in the transformer body). (C) Pipeline for deleteriousness prediction without external resources. MutFormer output Embeddings are obtained by feeding the input protein sequence (single or paired) to the MutFormer model. A fully connected layer is attached after this to obtain the resulting deleteriousness prediction. (D) Pipeline for deleteriousness prediction with external resources. MutFormer's output embedding and the sequence representation of all external predictions are concatenated, then a fully connected layer is used to obtain the final prediction.

Paired sequence classification. The input is a pair of two sequences: the mutated protein sequence and its corresponding reference sequence. The model classifies the aggregate of the sequences as deleterious or benign through a comparison of the two sequences. This was inspired by the sentence similarity problem (e.g., the MRPC[36] task) in NLP (Figure 2C).

Exploration of optimal fine-tuning methods

To find the best fine-tuning method, model, and hyperparameters, we performed two different internal comparison tests using our independent validation set. Test 1 compared the MutFormer architecture with the classical BERT architecture, as well as the three different fine-tuning methods, using different hyperparameters. Test 2 compared the use

Table 1. Model sizes of the pre-trained models (subscripts in the model names denote the number of self-attention layers)

Model name	Hidden layers	Hidden size	No. of parameters
$MutBERT_{8L}$	8	768	58M
$MutBERT_{10L}$	10	770	72M
$MutFormer_{8L}$	8	768	62M
$MutFormer_{10L}$	10	770	76M
$MutFormer_{12L}$	12	768	86M
$MutFormer_{8L\ (with\ integrated\ convs)}$	8	768	64M

$MutFormer_{12L}$ has the same size as $BERT_{Base}$. Hyperparameters constant for all models: intermediate size = 3072, maximum input sequence length: 1024.

of the integrated convolution implementation against the classic MutFormer architecture. Before both tests, some initial testing was done to establish a set of hyperparameter values that worked well with all combinations of methods/models included in the test. For test 1, the initial set of hyperparameters was established based on the three fine-tuning methods (per residue, single sequence, paired sequence) and three different models ($MutBERT_{8L}$, $MutBERT_{10L}$, and $MutFormer_{8L}$). For test 2, the initial set of hyperparameters was established based on the four models ($MutFormer_{8L}$, $MutFormer_{10L}$, $MutFormer_{12L}$, and $MutFormer_{8L\ (with\ integrated\ convolutions)}$) being tested. A full list of hyperparameters used is detailed in Table 3, and results of test 1 and test 2 are displayed, respectively, in Figures 3, 4, 5, S3, and S6.

Incorporation of external predictions

To create a model capable of the best possible performance in deleteriousness prediction, in addition to using protein sequence analysis, when training our final models (displayed in our final testing) (Figure 4), MutFormer also incorporated prediction values from previous methods published in literature. Computational predictions from previously published methods were given to MutFormer as input in the following way. First, using ANNOVAR, predicted scores for all mutations within a newly generated test set were obtained from the dbNSFPv3 database.[11] These scores were standardized between 1 and 2, and all missing predictions were assigned values of 0. A fully connected dense layer was connected to these inputs, and the output of this dense layer was concatenated with the original model output. Another dense layer after this concatenated result was then connected to the output node to produce the end prediction result (Figure 1D). This incorporation strategy prevents the model from becoming reliant on external predictions, limiting the weighting of sequence analysis vs. external predictions to about 1:1 in MutFormer's prediction (since the concatenated output of the two sequences of information is of the same length). To confirm this use ratio, upon completing fine-tuning of the various models, we analyzed the weights of both the combining dense layer, as well as the final output layer. When calculating the weighting sum over both layers, we found, for all models, an approximate weighting of 0.5 for MutFormer's sequence analysis, and 0.5 for external predictions. In particular, the fine-tuned model that represents MutFormer in Figures 4, 5, S3, and S6 has a weighting of 0.485 for sequence analysis and 0.515 for external predictions.

Testing MutFormer against existing methods of deleteriousness prediction

To assess the performance of MutFormer against existing methods of deleteriousness prediction, a total of five testing datasets were used. Out of these five datasets, three are non-gene-specific and non-disease-specific datasets, and two are gene-specific mutation datasets (details for each testing dataset used are outlined in Table 2). For each dataset, filtering was performed using the reference sequences to ensure that no bias was present: all mutations that shared reference sequences with any mutation present in the pre-training data were deleted from the testing sets, and all mutants with identical reference sequences present in any of the independent test sets were removed from the fine-tuning training data before model training.

To allow for a more comprehensive evaluation of the performance of MutFormer with different levels of "fit" on a wide range of data (models with a higher fit will perform better on more similar data, but worse on more dissimilar data; models with a lower fit will have the opposite tendencies), different MutFormer models with varying hyperparameters that affected a model's level of fit were trained (an analysis of MutFormer's performance with varying levels of fit is analogous to an analysis of a receiver operator characteristic (ROC)-type curve, where the performances of MutFormer on similar vs. dissimilar data is compared for different fit levels). In this test, the number of freezing layers and batch size were varied, while all other hyperparameters were set to the best ones found during our hyperparameter test 2 (see above). From these results, testing sets 3–5 showed more variation with different fit parameters than sets 1–2 did (results from all test runs are summarized in Figure S2). All models were then tested on all testing datasets, and the overall best-performing model across all testing datasets was used to represent MutFormer in our comparison. Batch sizes of 16, 32, and 64 were tested in conjunction with freezing layer numbers of 0, 5, 6, and 8 (full hyperparameter description in Table 3). Freezing layer numbers are defined as the number of transformer body layers that were frozen, starting from the first layer (for MutFormer 8L with integrated convolutions, our current best-performing model, eight layers is the total number of transformer body layers). For any freezing layer number greater than 0, the embedding layers were frozen as well (through testing on our validation set we found that leaving the embedding layers trainable while freezing the transformer layers significantly decreased performance). Each model was trained for 14k steps, and checkpoint steps 6k, 8k, 11k, 12k, and 14k were evaluated.

For each dataset, MutFormer is shown twice: once without the incorporation of other scores and only relying on sequence data alone (labeled as "MutFormer (no ext)" in Figures 4, 5, S3, and S6), and another with the use of external predictions as part of its input as described in the Incorporation of external predictions section (labeled as "MutFormer" in Figures 4, 5, S3, and S6). For MutFormer without the incorporation of other scores, like MutFormer with the incorporation of external scores, we tested various models with varying levels of fit, with an initial set of hyperparameters found through testing based on our independent validation set. The full set of hyperparameters for both of these MutFormer models is displayed in Table 3.

When fine-tuning our final models (used in our comparison of MutFormer vs. existing methods), to increase MutFormer's overall generalization ability and limit overfitting, data augmentation was implemented for the fine-tuning training data: for all epochs of data, each datapoint had a 50% chance of being altered. Those that were selected to be altered would be trimmed down to anywhere from 50% length of the original sequence to 100% length of the original sequence. Trimming was done around the mutation site to ensure the mutation site on average stayed in the same location (in the middle) in the sequence before and after trimming. Epochs were also shuffled independently of each other.

RESULTS

Effect of MutFormer's use of convolutions on the pre-training task

During pre-training, we trained models of different sizes for both the MutFormer architecture and MutBERT (MutFormer model without convolutions) architecture (Table 1). The loss and accuracy on the pre-training task are shown in Table S2. According to our results, the accuracy of $MutFormer_{8L}$ on the pre-training task test set was 54.5% higher than $MutBERT_{8L}$, indicating the advantage of the convolutions. In addition, the accuracy of $MutFormer_{8L}$ was 27.7% higher than that of $MutBERT_{10L}$, despite the latter having two more transformer layers and 10M more parameters (the subscript of each model name indicates only the number of transformer layers but does not consider the two convolutional layers). This outperformance despite its smaller size verifies that the improved performance of the model with convolutions was not simply due to the additional number of parameters or additional layers.

Performance of different fine-tuning methods and hyperparameters

As a part of our internal comparison test 1, we fine-tuned MutFormer and MutBERT using three methods: per residue classification, single sequence classification, and paired sequence classification (Figure 2; see Materials and methods for details). The ROC curves and corresponding area under the curve (AUC) for deleteriousness prediction are shown in Figures 3A–3C. Figure 3D shows a summary of the performance comparison of the three methods; paired sequence classification performed best, followed by per residue classification, and the optimal results were achieved by using a maximal input sequence length of 512 (use of the paired sequence method means an aggregate sequence length of 1,024) (Figure 3F). Upon examination of the performance of different model architectures, as shown in Figure 3E, $MutFormer_{8L}$ outperformed $MutBERT_{8L}$ and $MutBERT_{10L}$ for each fine-tuning method, indicating the advantage of the MutFormer architecture.

MutFormer's use of integrated convolutions

In our internal comparison test 2, two different strategies for implementing the convolutions were tested: classic MutFormer, and MutFormer (with

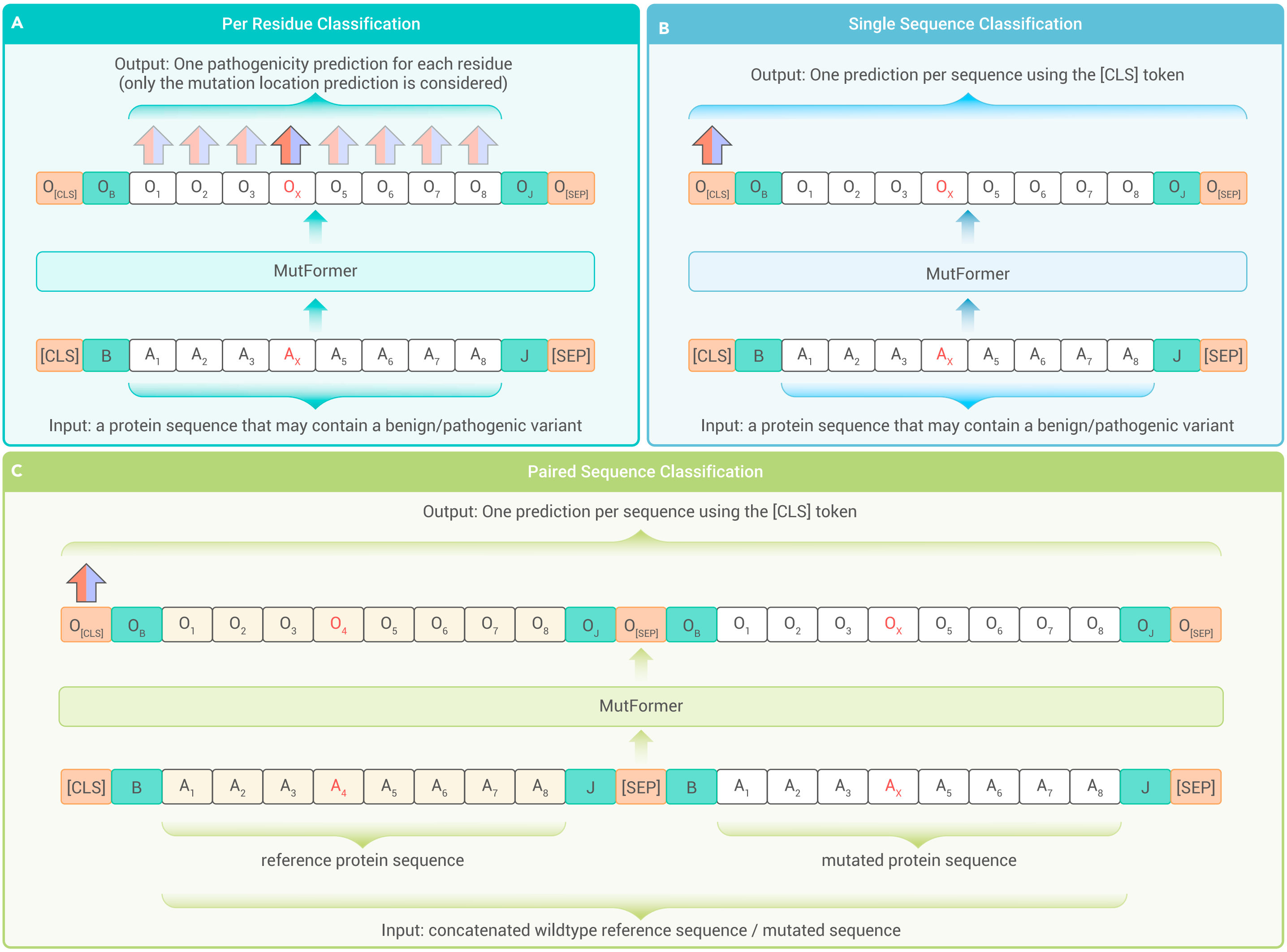

Figure 2. Different fine-tuning methods tested in this study (A) Per residue classification. The input is a protein sequence that contains exactly one variant. Each residue (amino acid) is given a label of benign/deleterious. Benign variants and residues that are identical to the reference sequence are labeled as benign. The fine-tuning task is to predict the label of each amino acid. This is similar to token classification problems (e.g., named entity recognition) in NLP. (B) Single sequence classification. The input is a protein sequence that contains exactly one variant with unknown significance. The embedding of the [CLS] token in the last layer is used to predict whether the sequence contains a deleterious variant. This is similar to sentence classification problems (e.g., sentiment analysis) in NLP. (C) Sequence pair classification. The input is a pair of two sequences: a reference protein sequence and a mutated protein sequence (with a benign or deleterious variant in the center). The embedding of the [CLS] token in the last layer is used to predict whether the mutated sequence contains a deleterious variant. This is similar to sentence pair classification problems (e.g., sentence similarity) in NLP.

integrated convolutions). Justification for the second implementation strategy is as follows: while the convolution mechanism should in theory be able to create a representation that will enable the model to best interpret the protein sequence, some information that is present in the original raw embedded sequence may be lost in practice through the convolutions. To solve this, the integrated convolutions model, instead of feeding the embeddings through the convolutions linearly, uses skip connections that result in the convolutions acting as an integrated part of the original embedding layers, allowing the transformer model to access both the convolution filtered representation of the sequence as well as the original embedded representation. In our internal comparison test 2, we compared the performance of MutFormer (with integrated convolutions) in paired sequence classification of our independent validation set to that of the other three original MutFormer architecture models (Figure S1). ROC curves are shown in Figure S1A, and a summary comparison histogram of the four different models tested is shown in Figure S1B. Overall, the margins of difference are small but, based on the results, the performance of the MutFormer model with integrated convolutions is higher than that of the original MutFormer model; even with only eight transformer layers, the integrated convolutions model outperformed MutFormer$_{12L}$, which had more layers and generally a better prediction ability than MutFormer$_{8L}$.

Comparison with existing variant prediction methods

As paired sequence classification performed best, for the comparison of MutFormer vs. other methods, this fine-tuning method was used. MutFormer's best overall performance was achieved by training our best model, MutFormer$_{8L\ (\text{with integrated convolutions})}$, on a batch size of 32, 0 freezing layers, and access to external predictions (for full hyperparameter descriptions, see Table 3). MutFormer's performance was compared against a variety of existing methods of deleteriousness prediction, including sequence alignment/homology-based scores (SIFT,[13] PolyPhen-2,[14] LRT,[39] MutationTaster,[40,41] MutationAssessor,[42] FATHMM,[43] PROVEAN,[44] phastCons,[45] and SiPhy[46]), ensembl-based scores (CADD,[16,19] MetaSVM,[17] MetaLR,[17] VEST3,[47] and DANN[48]), conservation based scores (GERP++,[15] PhyloP,[49] and fitCons[50]), we well as some other deep learning-based approaches (FATHMM-MKL,[51] VARITY,[52] and MVP[22]), out of which MVP and VARITY are recently developed methods. In our comparison, existing methods' predictions were processed in the following way (note that this differs from the incorporation strategy of external scores into MutFormer as input): each method's predictions were standardized from 0 to 1, based on prediction values of all possible missense mutations present in the dbNSFPv3 database.[11] Missing predictions were automatically assigned a prediction value of 0. Both non-inverted and inverted prediction identities (1 = deleterious, 0 = benign and 0 = deleterious, 1 = benign) were tested across our fine-tuning

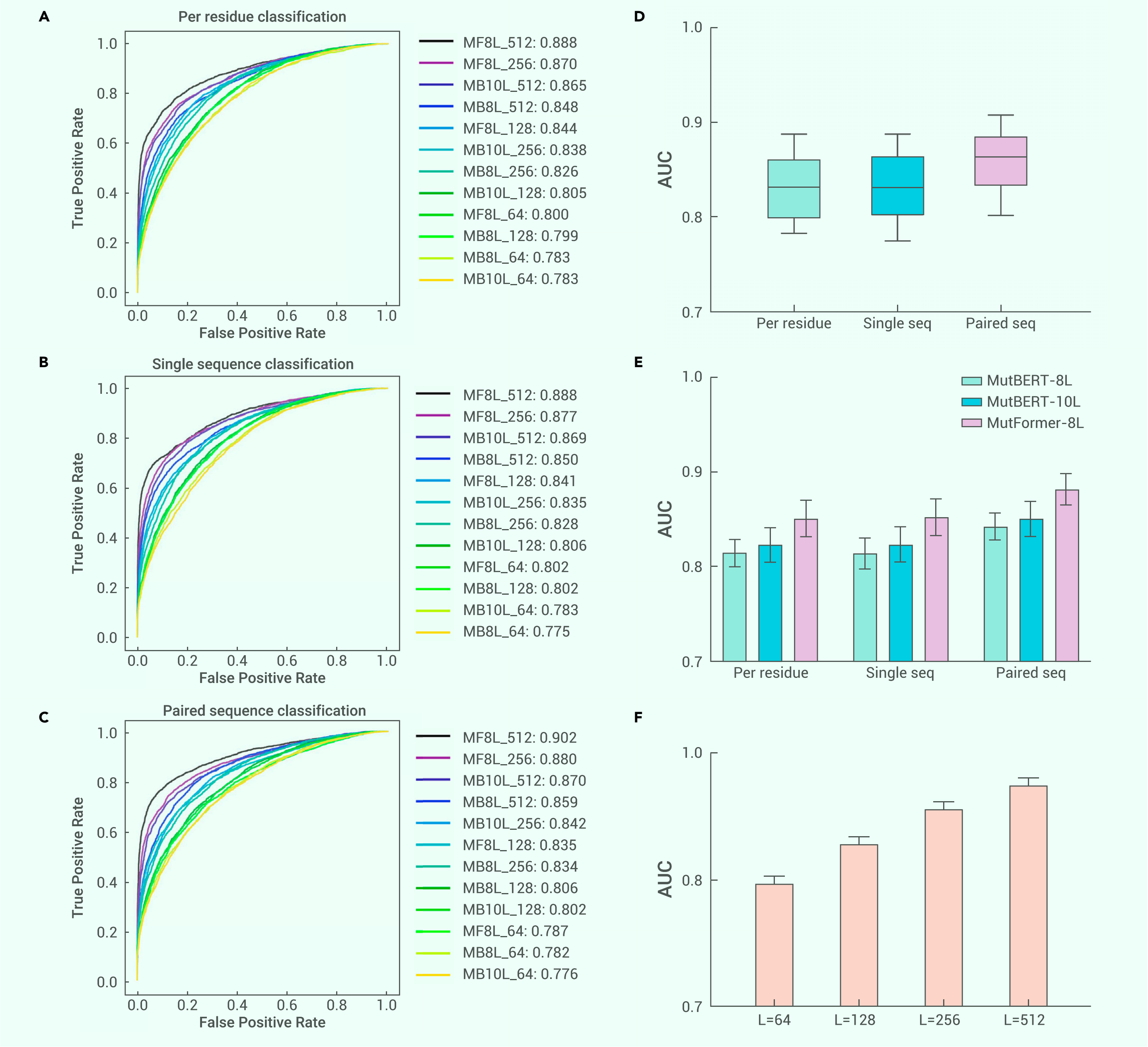

Figure 3. Exploration of optimal fine-tuning methods Performance comparison of different fine-tuning methods and MutFormer architecture/MutBERT architecture (internal comparison test 1). (A–C) ROC curves of performances on our independent validation set (8,427 data points total) for two different model architectures (MutFormer (without integrated convolutions) and MutBERT) and three fine-tuning methods (per residue, single sequence, paired sequence). Labels are in the following format: "[model short name]_[max input sequence length]: [AUC score]". Note that in (A–C), "MB" indicates MutBERT architecture and "MF" indicates MutFormer architecture. (D) Performance comparison of three different fine-tuning methods, using AUC scores shown in (A–C). Whiskers indicate minimum and maximum values. (E) Performance comparison of three pre-trained models: $MutBERT_{8L}$, $MutBERT_{10L}$, and $MutFormer_{8L}$. (F) Performance of different max input sequence lengths. (E, F) The results are mean ± SEM.

training data, and inversion of scores was done accordingly for each algorithm being compared. We generated both an ROC curve and, because of the unbalanced nature of some of our datasets, a precision-recall-gain curve for each dataset. For dataset 4, which only included rare benign examples, a threshold for each existing method, chosen by taking the point closest to the upper left corner on a ROC curve based on MutFormer's fine-tuning training data, was used to calculate a method specificity for each method. Upon analyzing the performances of the different testing datasets, we found that the best overall performing MutFormer model outperforms previous methods of deleteriousness prediction in non-gene-specific and non-disease-specific datasets (more similar to MutFormer's fine-tuning training dataset: sets 1–3). On the two gene-specific databases (sets 4–5), which contain data less similar to that of MutFormer's fine-tuning data, MutFormer's performance in comparison with other methods expectedly drops, while still matching the performance of various existing methods. For MutFormer without external predictions, its performance is first among non-MutFormer methods for sets 2 and 3, among the top for set 1, and drops further than MutFormer with incorporated external predictions for sets 4 and 5. ROC curves for all datasets are displayed in Figure 4, and precision-recall-gain curves of the same testing results for datasets 1, 2, 4, and 5 are displayed in Figure 5. A bar plot display of all ROC and PRG AUC values for datasets 1, 2, 4, and 5 is also presented in Figure S6. Only three methods ever have PRG AUC values below 0 (these values were clipped to 0 for the best display quality in the bar plot). All three of these instances occurred for dataset 4: fitCons had a PRG AUC performance of −0.312, phyloP7way_vertebrate −0.633, and phyloP20way_mammalian −0.293. Because of a large number of existing methods compared, we also used the Delong

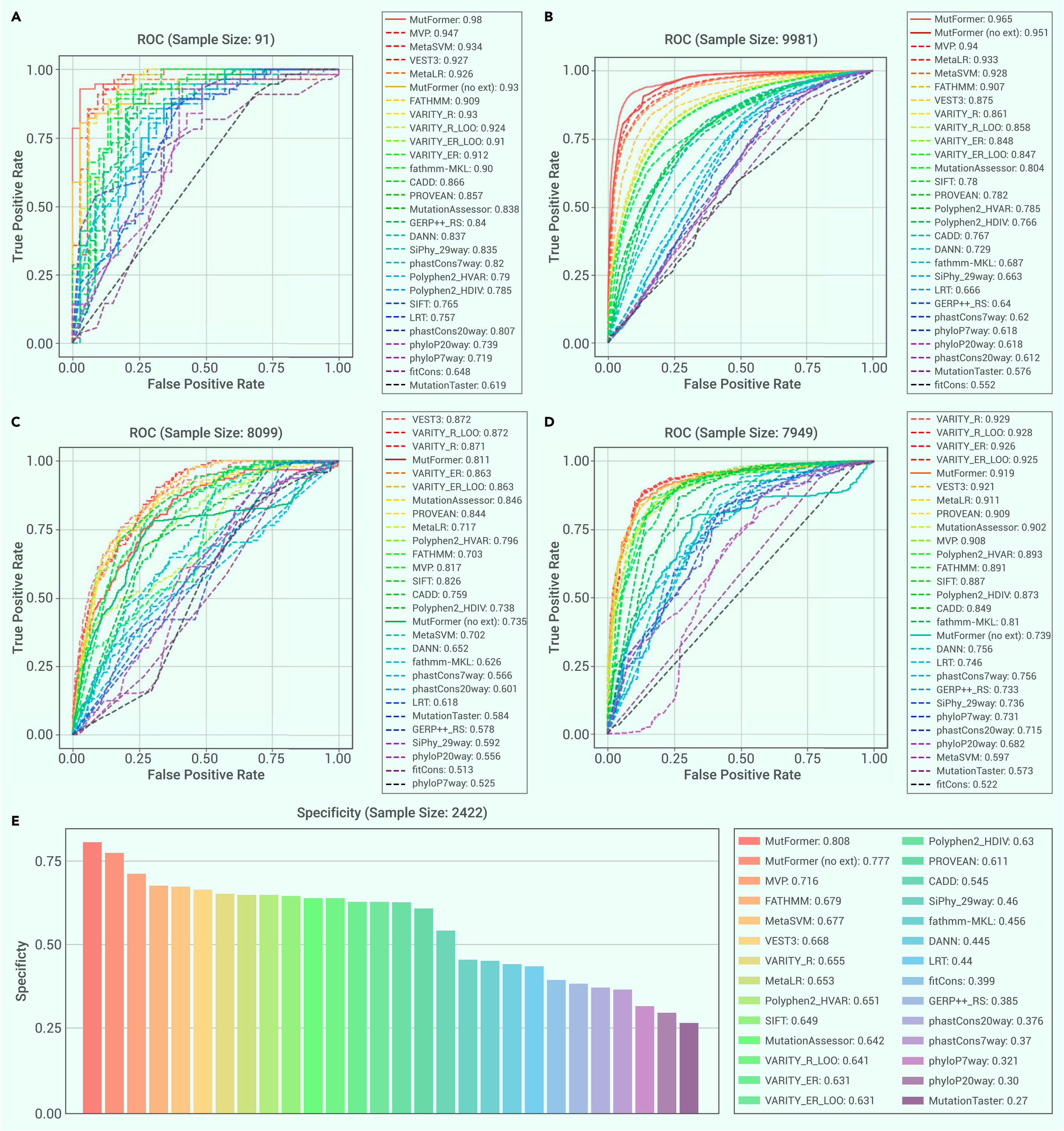

Figure 4. ROC curves for performance comparison with existing methods ROC curves and performance metrics of MutFormer and different existing methods of deleteriousness prediction evaluated on five different databases. The model which represents MutFormer here is MutFormer$_{8L\ \text{(with integrated convolutions)}}$, fine-tuned on a batch size of 32 with 0 freezing layers. Note that in this figure, "Mutformer" represents MutFormer's performance with the incorporation of external predictions, while "MutFormer (no ext)" represents Mutformer's performance without the use of external predictions. Labels are formatted in the following way: "[Method]: [Performance Metric]." (A) Meta_SVM_LR_set_1 – dataset compiled by a previous paper that originally outlined the MetaSVM and MetaLR methods, containing 56 negative examples and 35 positive examples. (B) MetaSVM_LR_set_2 – same source as Meta_SVM_LR_set_1, containing 5,866 negative examples and 4,115 positive examples. (C) Varibench_PPARG – dataset from Varibench for the peroxisome proliferator-activated receptor (gamma) gene, containing 4,671 negative examples and 3,428 positive examples. (D) Varibench_TP53 – dataset from Varibench for the TP53 gene, which codes for the tumor suppressor P53 protein, containing 3,444 negative examples and 4,505 positive examples. (E) MetaSVM_LR_set_3 – same source as Meta_SVM_LR_set_1 and set_2, containing 2,422 negative examples. Because only negative examples are present, ROC is invalid in this case; instead, specificity is used for comparison.

test of ROC to statistically assess the pairwise probability that the given methods' ROC curves were significantly different. Delong test results for datasets 1, 2, 4, and 5 are displayed in Figure S3.

Auxiliary tests

In addition to the results of the above tests, to further assess various aspects of MutFormer's strengths and weaknesses in various other areas, we have

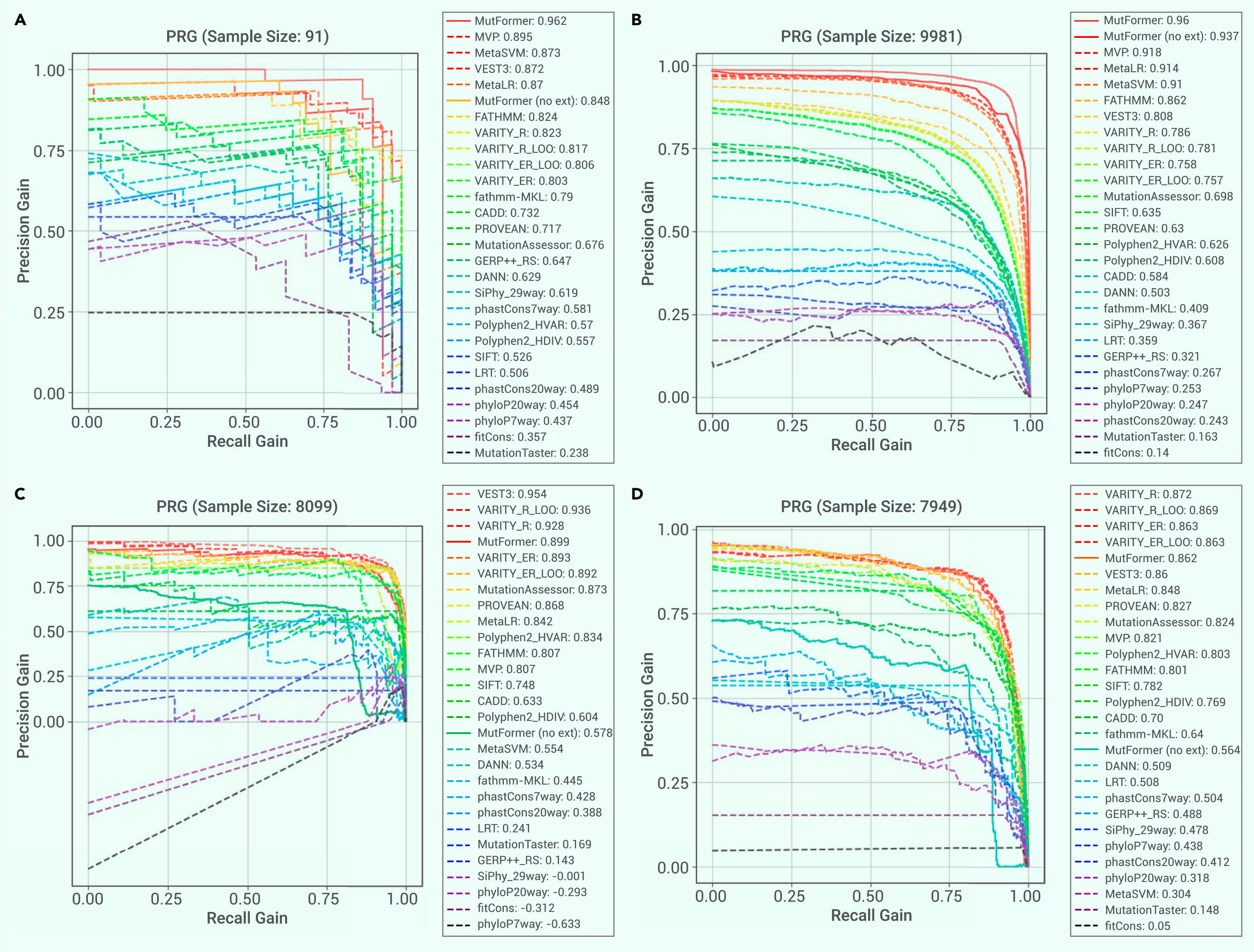

Figure 5. PRG curves for performance comparison with existing methods Precision-recall-gain (PRG) Curves/AUC scores of MutFormer and different existing methods of deleteriousness prediction for four out of our five testing datasets (MetaSVM_LR_set_3 is omitted in this case since PRG is invalid for data with only one class of true labels). The model, which represents MutFormer here is MutFormer$_{8L}$ (with integrated convolutions), fine-tuned with a batch size of 32 and 0 freezing layers. Note that "MutFormer" represents MutFormer with the incorporation of external predictions, while "MutFormer (no ext)" represents MutFormer without the use of external predictions. Labels are formatted in the following way: [Method]: [AUC score]. (A) Meta_SVM_LR_set_1. (B) MetaSVM_LR_set_2. (C) Varibench_PPARG. (D) Varibench_TP53.

performed five additional studies, each detailed in the Supplemental Methods: bias analysis in Mutformer's fine-tuning data, an ablation study on MutFormer's use of external predictions, an assessment of MutFormer's correlation with existing evolutionary methods, an evaluation of MutFormer's performance on the ProteinGym dataset, and an analysis of MutFormer's weights for biological significance.

Precomputed deleteriousness scores for all missense mutations

To facilitate future use by other studies, we precomputed deleteriousness scores for all missense mutations using the best-performing MutFormer model. The inference was done on a cloud TPU device (v2-8), which took approximately 11.5 h. These scores can be directly used in the ANNOVAR software to annotate missense variants identified from genome or exome sequencing, and are organized in a flat-file format, allowing for easy use in other functional annotation software tools to complement the dbNSFP database, which has a variety of other prediction scores for missense mutations in the human genome.

DISCUSSION

In the current study, we present MutFormer, a transformer-based machine-learning model to predict the deleteriousness of non-synonymous SNVs using protein sequence as the primary feature. We pre-trained MutFormer on reference protein sequences and alternative protein sequences resulting from common genetic variants in the human genome and tested different fine-tuning methods for deleteriousness prediction. During our evaluation processes, MutFormer outperformed multiple commonly used methods and had comparable performances with other methods even when tested on datasets that were less similar to MutFormer's training data (gene-specific data, sets 5 and 6). Below we discuss several advantages and limitations of the MutFormer method and its computational package.

Although a large number of computational tools have been developed over the years on predicting the deleteriousness of non-synonymous mutations, to the best of our knowledge, MutFormer is among the first batch of tools that utilize transformer models to adapt the biological sequence analysis problem as a language analysis problem. A similar model of note is ProtBERT,[53] a previous application of the BERT architecture to protein contexts. Despite both using the transformer architecture, MutFormer differs from ProtBERT in several key aspects: (1) MutFormer makes use of convolutions to learn its own vocabulary, while ProtBERT uses a fixed vocabulary; (2) MutFormer was pre-trained on human protein sequences and common variants, while ProtBERT was trained on reference sequences of all species with sequence information; and (3) MutFormer's primary goal was deleteriousness prediction while ProtBERT focused on subcellular localization of proteins and secondary structure prediction. ConvBERT[54] is another method that like MutFormer, uses a combination of CNNs and the transformer architecture. However, MutFormer

Table 2. Details for each testing dataset

Testing dataset	Testing dataset composition	Compilation year
Set 1: Meta_SVM_LR_set_1	Dataset compiled by a previous method, Meta_SVM/Meta_LR. Used to assess Meta_SVM and Meta_LR's performance against other methods.<br>Composition after filtering:<br>● 56 pathogenic examples compiled from recent Nature Genetics publications at the time.<br>● 35 benign examples from the CHARGE (Cohorts for Heart and Aging Research in Genetic Epidemiology) database, which focuses on identifying genes underlying heart, lung, and blood diseases.	2015
Set 2: Meta_SVM_LR_set_2	Dataset from the same source as set 2 (Meta_SVM_LR_set_1)<br>Composition after filtering:<br>● 4,135 pathogenic examples from Varibench testing dataset II for missense mutations[37] (Varibench is a dataset designed specifically for the testing of prediction methods for pathogenicity).<br>● 5,884 benign examples also from Varibench testing dataset II.	2015
Set 3: Meta_SVM_LR_set_3	Dataset from the same source as set 2 (Meta_SVM_LR_set_1) set 3 (Meta_SVM_LR_set_2).<br>Composition after filtering:<br>● 2,422 benign examples from the CHARGE database.	2015
Set 4: Varibench_PPARG	Dataset from Varibench.[37] Compiled by a study that specifically aimed to create datasets for assessing computational models' performance in pathogenicity prediction of missense mutations.[38] Focused on the PPARG gene which codes for the gamma member of the PPAR (Peroxisome Proliferator-activated Receptor) family of nuclear receptors, which can be linked to the pathology of diseases including diabetes, atherosclerosis, and cancer.<br>Composition after filtering:<br>● 145 pathogenic variants from the experimentally validated Missense InTerpretation by Experimental Response (MITER) database.<br>● 2,207 benign variants from the same source.	2018
Set 5: Varibench_TP53	Dataset from same source as Set 5 (Varibench_PPARG). Focused on the TP53 gene which codes for tumor protein p53, a tumor suppressor gene.<br>Composition after filtering:<br>● 608 pathogenic examples from the IARC database (database specific for TP53), labeled for significantly changing the gene expression level of the TP53 gene.<br>● 531 benign examples also from IARC which did not change expression level significantly.	2018

and ConvBERT differ both in their architectural incorporation of convolutions as well as their motivation for using convolutional layers. The primary motivation for the use of convolutions in Mutformer is that the tokenization of protein sequences into individual residues does not necessarily represent the breaking of a given sequence into individual residues (the true residues of proteins are more accurately amino acid motifs within the protein's 3D structure). For MutFormer, the convolutions are placed as part of the embedding layers to create more useful and efficient representations of a given sequence. In contrast, regular NLP tasks, such as those which ConvBERT attempts to address, do not have such problems (words are generally more or less residues in natural language). Instead, ConvBERT's architecture uses convolutions within the attention module, with the motivation being the use of convolutions as a way to efficiently capture short-range dependencies.

In addition, the training process of MutFormer is simple and straightforward. MutFormer uses a self-supervised pre-training strategy and therefore does not require any labeled data. For this reason, a large model with hundreds of millions of parameters can be trained on a large amount of non-curated data. On this note, while the current study focused on the human genome exclusively, it is conceivable to include other well-annotated genomes from other species in future studies to see whether increased complexity in the sequence space during pre-training can further improve performance. In the fine-tuning stage, MutFormer learns the deleteriousness of mutations based on the labeled training data as well as its understanding of protein sequence already learned in the pre-training stage, allowing a small amount of fine-tuning data to be effectively used to achieve an accurate result. Furthermore, transformers consider attention, which is not only useful for understanding context in language processing problems, but could also give important insights into the deleteriousness effects that amino acids can have under different sequence contexts. Even though direct feature attribution is not possible with a model containing millions of intertwined model parameters such as MutFormer, our analyses of MutFormer's model weights show that MutFormer is able to prioritize and model these relationships through the use of convolutions and attention.

There are also several limitations of the current study. First, the training data and testing datasets are still of limited size, and testing on large-scale experimentally or clinically supported datasets would result in a more effective evaluation of usability. In the future, MutFormer can be evaluated on large-scale genome sequencing data followed by manual review, to determine whether it helps to prioritize deleterious variants in clinical sequencing settings. Second, because of computational limitations, we did not fully test all parameters during training. As a result, it is likely that our results are not completely optimized; larger models using longer maximum sequence lengths would also be able to outperform the current MutFormer models (e.g., a 12-layer MutFormer with integrated convolutions should perform noticeably better than the best current MutFormer model with only eight attention layers). Third, in the deleteriousness prediction of missense mutations, it is likely impossible for a given model to obtain all required evidence from sequence data alone, so incorporation of other features, such as a three-dimensional (3D) structure (i.e., analyzing 3D structure to scale attention with 3D distance, or labeling sections as belonging to beta sheets or alpha helices for better prediction of deleteriousness), methylation, clinical phenotypic information (i.e., using this knowledge to prioritize certain genes), and other features that could significantly affect proteins' behavior, could reasonably improve overall understanding and thus performance.

In summary, MutFormer is a novel transformer-based method to predict the functional effects of missense mutations. We hope that MutFormer can bring new insights to the bioinformatics community, by being a language model capable of improving our understanding of the language of proteins. Given that MutFormer used complementary information that other bioinformatics tools developed for deleteriousness prediction, we also envision that they could be combined to reach consensuses on predictions, which may be useful in implementation into current clinical guidelines.

DATA AND CODE AVAILABILITY

The source code to run MutFormer, all six pre-trained models, a reproducible workflow, and the best-performing fine-tuned models are available at the GitHub repository: https://github.com/WGLab/MutFormer.

Table 3. MutFormer Fine-tuning hyperparameter specifications

Test/model	Model architecture	Fine-tuning method	Initial/end learning rate	Training steps
Internal comparison test 1	(MutFormer$_{8L}$, MutBERT$_{8L}$, MutBERT$_{10L}$)	(Per residue, single sequence, paired sequence)	1e-5/(2e-7 to 1e-6)	(4k [fine-tuning method 1 and 2], 10k [fine-tuning method 3])
Internal comparison test 2	(MutFormer$_{8L}$, MutFormer$_{10L}$, MutFormer$_{12L}$, MutFormer$_{8L}$ (with integrated convs)	paired sequence	1e-5/1.4e-6	12k
MutFormer comparison with others	MutFormer$_{8L}$ (with integrated convs)	paired sequence	1e-5/3e-9	14k (evaluated on 6k, 8k, 11k, and 12k)
MutFormer final model (with external predictions)	MutFormer$_{8L}$ (with integrated convs)	paired sequence	1e-5/3e-9	12k
MutFormer final model (no external predictions)	MutFormer$_{8L}$ (with integrated convs)	paired sequence	1e-5/3e-9	8k

Test/model	Max input sequence length	Batch size	Weight decay	Freezing layers	External predictions
Internal comparison test 1	(64, 128, 256, 512)	16	0.01	0	no
Internal comparison test 2	(256, 512)	(16, 32, 64)	0.01	0	no
MutFormer comparison with others	512	(16, 32, 64)	0.01	(0, 5, 6, 8)	yes
MutFormer final model (with external predictions)	512	32	0.01	0	yes
MutFormer final model (no external predictions)	512	32	0	0	no

Additional hyperparameters constant for all runs: - Gradient clip amount: No gradient clipping was used during fine-tuning (this was used previously during pre-training).

REFERENCES

1. 1000 Genomes Project Consortium, Auton, A., Brooks, L.D., Durbin, R.M., et al. (2015). A global reference for human genetic variation. Nature ***526***, 68–74.
2. Stenson, P.D., Mort, M., Ball, E.V., et al. (2020). The Human Gene Mutation Database (HGMD®): optimizing its use in a clinical diagnostic or research setting. Hum. Genet. ***139***, 1197–1207.
3. Lek, M., Karczewski, K.J., Minikel, E.V., et al. (2016). Analysis of protein-coding genetic variation in 60,706 humans. Nature ***536***, 285–291.
4. Karczewski, K.J., Weisburd, B., Thomas, B., et al. (2017). The ExAC browser: displaying reference data information from over 60 000 exomes. Nucleic Acids Res. ***45***, D840–D845.
5. Karczewski, K.J., Francioli, L.C., Tiao, G., et al. (2020). The mutational constraint spectrum quantified from variation in 141,456 humans. Nature ***581***, 434–443.
6. Landrum, M.J., Lee, J.M., Riley, G.R., et al. (2014). ClinVar: public archive of relationships among sequence variation and human phenotype. Nucleic Acids Res. ***42***, D980–D985.
7. Landrum, M.J., Lee, J.M., Benson, M., et al. (2018). ClinVar: improving access to variant interpretations and supporting evidence. Nucleic Acids Res. ***46***, D1062–D1067.
8. Landrum, M.J., Chitipiralla, S., Brown, G.R., et al. (2020). ClinVar: improvements to accessing data. Nucleic Acids Res. ***48***, D835–D844.
9. Liu, X., Li, C., Mou, C., et al. (2020). dbNSFP v4: a comprehensive database of transcript-specific functional predictions and annotations for human nonsynonymous and splice-site SNVs. Genome Med. ***12***, 103.
10. Liu, X., Jian, X., and Boerwinkle, E. (2011). dbNSFP: A lightweight database of human nonsynonymous SNPs and their functional predictions. Hum. Mutat. ***32***, 894–899.
11. Liu, X., Wu, C., Li, C., et al. (2016). dbNSFP v3.0: A One-Stop Database of Functional Predictions and Annotations for Human Nonsynonymous and Splice-Site SNVs. Hum. Mutat. ***37***, 235–241.
12. Thusberg, J., Olatubosun, A., and Vihinen, M. (2011). Performance of mutation pathogenicity prediction methods on missense variants. Hum. Mutat. ***32***, 358–368.
13. Ng, P.C., and Henikoff, S. (2003). SIFT: Predicting amino acid changes that affect protein function. Nucleic Acids Res. ***31***, 3812–3814.
14. Adzhubei, I.A., Schmidt, S., Peshkin, L., et al. (2010). A method and server for predicting damaging missense mutations. Nat. Methods ***7***, 248–249.
15. Davydov, E.V., Goode, D.L., Sirota, M., et al. (2010). Identifying a high fraction of the human genome to be under selective constraint using GERP++. PLoS Comput. Biol. ***6***, e1001025.
16. Kircher, M., Witten, D.M., Jain, P., et al. (2014). A general framework for estimating the relative pathogenicity of human genetic variants. Nat. Genet. ***46***, 310–315.
17. Dong, C., Wei, P., Jian, X., et al. (2015). Comparison and integration of deleteriousness prediction methods for nonsynonymous SNVs in whole exome sequencing studies. Hum. Mol. Genet. ***24***, 2125–2137.
18. Ioannidis, N.M., Rothstein, J.H., Pejaver, V., et al. (2016). REVEL: An Ensemble Method for Predicting the Pathogenicity of Rare Missense Variants. Am. J. Hum. Genet. ***99***, 877–885.
19. Rentzsch, P., Witten, D., Cooper, G.M., et al. (2019). CADD: predicting the deleteriousness of variants throughout the human genome. Nucleic Acids Res. ***47***, D886–D894.
20. Richards, S., Aziz, N., Bale, S., et al. (2015). Standards and guidelines for the interpretation of sequence variants: a joint consensus recommendation of the American College of Medical Genetics and Genomics and the Association for Molecular Pathology. Genet. Med. ***17***, 405–424.
21. Zhou, J., and Troyanskaya, O.G. (2015). Predicting effects of noncoding variants with deep learning-based sequence model. Nat. Methods ***12***, 931–934.
22. Qi, H., Zhang, H., Zhao, Y., et al. (2021). MVP predicts the pathogenicity of missense variants by deep learning. Nat. Commun. ***12***, 510.
23. Dunham, A.S., Beltrao, P., and AlQuraishi, M. (2023). High-throughput deep learning variant effect prediction with Sequence UNET. Genome Biol. ***24***, 110.
24. Devlin, J., Chang, M.-W., Lee, K., et al. (2019). BERT: Pre-training of Deep Bidirectional Transformers for Language Understanding. Preprint at ArXiv. https://arxiv.org/abs/1810.04805.
25. Vaswani, A., Shazeer, N.M., Parmar, N., et al. (2017). Attention is All you Need. Preprint at ArXiv. https://arxiv.org/abs/1706.03762.
26. Liu, Y., Ott, M., Goyal, N., et al. (2019). RoBERTa: A Robustly Optimized BERT Pretraining Approach. Preprint at ArXiv. https://arxiv.org/abs/1907.11692.
27. Lan, Z., Chen, M., Goodman, S., et al. (2019). ALBERT: A Lite BERT for Self-supervised Learning of Language Representations. Preprint at ArXiv. https://arxiv.org/abs/1909.11942.
28. Yang, Z., Dai, Z., Yang, Y., et al. (2019). XLNet: Generalized Autoregressive Pretraining for Language Understanding. Preprint at ArXiv. https://arxiv.org/abs/1909.11942.
29. Dosovitskiy, A., Beyer, L., Kolesnikov, A., et al. (2020). An Image is Worth 16x16 Words: Transformers for Image Recognition at Scale. Preprint at ArXiv. https://arxiv.org/abs/2010.11929.
30. Jumper, J., Evans, R., Pritzel, A., et al. (2021). Highly accurate protein structure prediction with AlphaFold. Nature ***596***, 583–589.
31. Avsec, Ž., Agarwal, V., Visentin, D., et al. (2021). Effective gene expression prediction from sequence by integrating long-range interactions. Nat. Methods ***18***, 1196–1203.
32. Wu, Y., Schuster, M., Chen, Z., et al. (2016). Google's Neural Machine Translation System: Bridging the Gap between Human and Machine Translation. Preprint at ArXiv. https://arxiv.org/abs/1609.08144.
33. Xue, L., Barua, A., Constant, N., et al. (2021). ByT5: Towards a token-free future with pre-trained byte-to-byte models. Preprint at ArXiv. https://arxiv.org/abs/2105.13626.
34. Duman Keles, F., Mahesakya Wijewardena, P., and Hegde, C. (2022). On The Computational Complexity of Self-Attention. Preprint at ArXiv. https://arxiv.org/abs/2209.04881.
35. Wang, K., Li, M., and Hakonarson, H. (2010). ANNOVAR: functional annotation of genetic variants from high-throughput sequencing data. Nucleic Acids Res. ***38***, e164.
36. Dolan, W.B., and Brockett, C. (2005). Automatically Constructing a Corpus of Sentential Paraphrases. In Proceedings of the Third International Workshop on Paraphrasing (IWP2005) https://aclanthology.org/I05-5002.
37. Sasidharan Nair, P., and Vihinen, M. (2013). VariBench: a benchmark database for variations. Hum. Mutat. ***34***, 42–49.
38. Li, J., Zhao, T., Zhang, Y., et al. (2018). Performance evaluation of pathogenicity-computation methods for missense variants. Nucleic Acids Res. ***46***, 7793–7804.

39. Chun, S., and Fay, J.C. (2009). Identification of deleterious mutations within three human genomes. Genome Res. **19**, 1553–1561.
40. Schwarz, J.M., Cooper, D.N., Schuelke, M., et al. (2014). MutationTaster2: mutation prediction for the deep-sequencing age. Nat. Methods **11**, 361–362.
41. Schwarz, J.M., Rödelsperger, C., Schuelke, M., et al. (2010). MutationTaster evaluates disease-causing potential of sequence alterations. Nat. Methods **7**, 575–576.
42. Reva, B., Antipin, Y., and Sander, C. (2011). Predicting the functional impact of protein mutations: application to cancer genomics. Nucleic Acids Res. **39**, e118.
43. Shihab, H.A., Gough, J., Cooper, D.N., et al. (2013). Predicting the functional, molecular, and phenotypic consequences of amino acid substitutions using hidden Markov models. Hum. Mutat. **34**, 57–65.
44. Choi, Y., Sims, G.E., Murphy, S., et al. (2012). Predicting the functional effect of amino acid substitutions and indels. PLoS One **7**, e46688.
45. Siepel, A., Bejerano, G., Pedersen, J.S., et al. (2005). Evolutionarily conserved elements in vertebrate, insect, worm, and yeast genomes. Genome Res. **15**, 1034–1050.
46. Garber, M., Guttman, M., Clamp, M., et al. (2009). Identifying novel constrained elements by exploiting biased substitution patterns. Bioinformatics **25**, i54–i62.
47. Carter, H., Douville, C., Stenson, P.D., et al. (2013). Identifying Mendelian disease genes with the variant effect scoring tool. BMC Genom. **14** (*Suppl 3*), S3.
48. Quang, D., Chen, Y., and Xie, X. (2015). DANN: a deep learning approach for annotating the pathogenicity of genetic variants. Bioinformatics **31**, 761–763.
49. Pollard, K.S., Hubisz, M.J., Rosenbloom, K.R., et al. (2010). Detection of nonneutral substitution rates on mammalian phylogenies. Genome Res. **20**, 110–121.
50. Gulko, B., Hubisz, M.J., Gronau, I., et al. (2015). A method for calculating probabilities of fitness consequences for point mutations across the human genome. Nat. Genet. **47**, 276–283.
51. Shihab, H.A., Rogers, M.F., Gough, J., et al. (2015). An integrative approach to predicting the functional effects of non-coding and coding sequence variation. Bioinformatics **31**, 1536–1543.
52. Wu, Y., Liu, H., Li, R., et al. (2021). Improved pathogenicity prediction for rare human missense variants. Am. J. Hum. Genet. **108**, 2389.
53. Elnaggar, A., Heinzinger, M., Dallago, C., et al. (2022). ProtTrans: Towards Cracking the Language of Lifes Code Through Self-Supervised Deep Learning and High Performance Computing. IEEE Trans. Pattern Anal. Mach. Intell. **44**, 7112–7127.
54. Jiang, Z.-H., Yu, W., Zhou, D., et al. (2020). Convbert: Improving bert with span-based dynamic convolution. Adv. Neural Inf. Process. Syst. **33**, 12837–12848.

ACKNOWLEDGMENTS

We acknowledge the TPU Research Cloud (TRC) program by Google, which provided us with TPUs for the majority of the project. The study is in part supported by NIH grant GM132713 (K.W.), the CHOP Research Institute and the Fundamental Research Funds for the Central Universities, Sun Yat-sen University (No. 23ptpy119, to L.F).

AUTHOR CONTRIBUTIONS

L.F. and K.W. conceived and guided the study. T.J. performed the study, developed the software tools, and wrote the manuscript. All authors have given final approval for the manuscript to be published and have agreed to be responsible for all aspects of the manuscript.

DECLARATION OF INTERESTS

The authors declare no competing interests.

SUPPLEMENTAL INFORMATION

It can be found online at https://doi.org/10.1016/j.xinn.2023.100487.

LEAD CONTACT WEBSITE

Kai Wang: https://wglab.org.

The Innovation, Volume *4*

Supplemental Information

Deciphering “the language of nature”: A transformer-based language model for deleterious mutations in proteins

Theodore T. Jiang, Li Fang, and Kai Wang

The Innovation, Volume ■■

Supplemental Information

Deciphering "the language of nature": A transformer-based language model for deleterious mutations in proteins

Theodore T. Jiang, Li Fang, and Kai Wang

Supplemental Methods

1. Data Preparation

1.1 Pretraining data generation

We pretrained MutFormer on human reference protein sequences (all isoforms) and protein sequences caused by non-synonymous SNVs with > 1% population frequency in the gnomAD database.[1] The 1% threshold was used to ensure that this collection of examples contained a higher percentage of truly benign variants since it is important for the model to learn the "syntax" of the language from "correct" examples during pretraining. The total number of protein sequences used during pretraining was 128,670. The maximum input length of MutFormer was set to 1024, where protein sequences longer than 1024 are cut into non-overlapping segments starting with the first residue (i.e. the first segment contains residues 1-1024). Before cutting, a "B" letter was added to the beginning of a sequence, and a "J" letter was added to the end of a sequence so that the true start and end of a protein sequence were also indicated ("B" and "J" are not included in the current biological amino acid alphabet). During cutting, all segments were retained, except for segments less than 50 amino acids long, which were discarded. In total, we accumulated 150,533 training data points from these 128,670 protein sequences, of which 86,213 were left intact when compared toc their original protein sequence (with both "B" and "J" tokens), 23,857 contained a "B" token but not a "J" token, 21,672 contained a "J" token but not a "B" token, 18,791 contained neither a "B" nor "J" token, and 2,561 original protein sequences did not have its tail end represented (because the tail end of the sequence was less than 50 amino acids long).

1.2 Training and evaluation data generation for variant deleteriousness prediction

We obtained 84K manually annotated pathogenic missense SNVs from the Human Gene Mutation Database (HGMD, version 2016).[2] We combined this set with SNPs from the gnomAD database[1] with allele frequency >0.1%, the vast majority of which are assumed to be benign. Although a commonly used allele frequency threshold for benign variants is 1%, we used 0.1% instead, in order to achieve 1) the exclusion of pretraining data from the fine-tuning data (MutFormer's pretraining data utilized the threshold of 1%); 2) the approximate balancing of the number of benign and deleterious/pathogenic examples in the dataset. Any mutations that appeared in both the HGMD database and benign set were removed, and from the remaining data, all the mutations present in the original pretraining data (i.e., >1% in gnomAD) were also eliminated. Within the training data, mutated sequences were obtained by mutating a reference protein sequence (using ANNOVAR) based on a nucleotide substitution specified by each variant in the dataset. In situations where the reference sequence residue did not match the reported reference residue by the mutation, the example was discarded. The final benign set contained 61K variants. The combination of the deleterious set and the benign set was randomly split into a training set and an independent validation set. The independent validation set was then isolated from the training set by reference sequence: to prevent memorization from the training set to the validation set, mutations were deleted from the training set if the mutated sequence or the mutation's reference sequence was present in the validation set. The independent validation set contains 5,282 benign variants and 3,145 deleterious variants. Note that this independent validation set, despite its independent selection, is still prone to bias because of its similarity with the training set. For this reason, this set is only used to internally compare the performance of MutFormer models trained within this study, and separate testing sets compiled from various different sources are used for the comparison of MutFormer with other existing methods of deleteriousness prediction.

2. Auxiliary Tests

2.1 Analyzing bias present in MutFormer's fine-tuning data

To gauge the amount of bias present in the resulting fine-tuning dataset, distributions of positive and negative examples per protein reference id are displayed in Figure S4A. However, since certain proteins are naturally

more sensitive to mutations than others, in order to appropriately interpret the positive and negative example distributions in the context of the human population, we graphed the probability of loss of function intolerance (pLI) score, obtained from GnomAD data for each corresponding protein ID in Figure S4B. From the two graphs, we see that protein IDs with relatively low pLI scores tend to have less deleterious representation and more benign representation in our fine-tuning dataset, suggesting that there is little bias introduced.

2.2 Ablation Study on MutFormer's Use of External Predictions

In order to assess the extent of MutFormer's use of each external prediction during pathogenicity prediction, we performed an ablation study for our best-performing model, MutFormer_{8L} with integrated convolutions (full hyperparameter description in Table 3), which represents MutFormer in our comparison with existing methods. The ablation study is outlined as follows: For each external prediction, we remove it from the input while keeping all other predictions constant. The performance of the model on our independent validation set with the method removed is then recorded. As can be seen from Figure S7, the removal of MVP as a source of information for MutFormer results in a noticeably larger drop in performance than other methods. This can perhaps be explained by MVP's consistently high performance on our independent testing datasets, making it a good source of information for MutFormer. Figure S7 displays the graph resulting from this ablation study.

2.3 MutFormer Complementing Evolutionary Approaches

Due to MutFormer's primary reliance on protein sequence data analysis, we hypothesized that MutFormer would be capable of providing complementary information to existing evolutionarily based methods for variant classification. To assess this, we compared MutFormer (external prediction included) to EVE[3] using the provided scores and prediction data from EVE's database. Only examples with reported clinical significance of benign, pathogenic, likely pathogenic, or likely benign were used. We calculated the Spearman Rank Correlation between MutFormer and EVE for mutations predicted by both MutFormer EVE. We found rank correlations of 0.320 and 0. 336 (both with p values less than $1\text{x}10^{-10}$) between MutFormer and EVE_ASM and between MutFormer and EVE_BPU, respectively. This rank correlation indicates that while MutFormer is correlated with EVE, the information provided by MutFormer is not identical to that which EVE provides, suggesting that MutFormer's ability to analyze sequences provides additional information to the protein interpretation problem which evolutionary approaches may not be capable of extracting.

2.4 ProteinGym Evaluation

In the interest of further assessing MutFormer's interpretation ability of protein sequences, we evaluated MutFormer's performance on Deep Mutational Scanning (DMS) data, which corresponds to a prediction of mutation fitness. Mutation evolutionary fitness is a task that is related to pathogenicity, and some methods natively used for predicting evolutionary fitness have been directly ported to pathogenicity prediction. Because evolutionary fitness prediction is not a task that MutFormer is directly optimized for, good performance on DMS data would further indicate MutFormer's general protein comprehensive ability. Using the ProteinGym database provided by Tranception,[4] we compared (external predictions included) MutFormer's performance on this set against all other methods provided by the ProteinGym database. Because some amino acid substitutions present in ProteinGym are not obtainable through SNPs, these amino acid substitutions were not included in our evaluation. After eliminating these examples from all methods' data, we used ProteinGym's evaluation script to run an evaluation of all methods alongside Mutformer on the remaining DMS data. As can be seen from Table S4, MutFormer is outperformed by various methods in the ProteinGym evaluation dataset. We observe that MutFormer, despite being optimized for pathogenicity prediction and not fitness prediction, still shows comparable performance with various current methods in this dataset. The full results of this evaluation are displayed in Table S4.

2.5 Analyzing the weights of MutFormer

The MutFormer model takes advantage of both convolutions and attention, both of which contain useful information about MutFormer's interpretation mechanisms for protein sequences. To better understand these mechanisms, we analyzed MutFormer's model weights for the final best-performing MutFormer model. For our analysis of the convolution layers, two visualizations were generated. In our first visualization, we plotted the average convolution filter weights for each of the four convolution operations. In our second visualization, based on each of the four convolution operations, we plotted the overall percentage consideration given to each input residue id (amino acid identity/special tokens). Both of these visuals are displayed in Figure S5A. Based on visual 2, we see that the overall considerations placed on each residue are very nearly identical across the four different convolutions. Special tokens, as expected, were given very large weight in overall consideration. Interestingly, the "J" token was given a relatively significant weighting, but the "B" token was not as relatively prioritized. Of note, amino acids Serine (S), Cysteine (C), and Valine (V) took more priority relative to other amino acids. Based on visual 1, we can see that first, there are different prioritizations of positions for each of the four convolutions, and additionally, that while specific patterns cannot be directly derived from these filters, it is reasonable to infer that combinations of residue IDs and residue ID patterns are learned by different convolutions.

For our analysis of attention weights, we opted for two case studies from the PDB database: PDB: 1B1C and PDB: 1P4O. The chosen proteins were selected because of their relatively large number of deleterious mutations and complex structure. We ran MutFormer with each protein sequence as input and generated four different visuals: 1) all 8 individual attention maps for each attention layer, 2) a rollout attention map representing the log scale result from taking the dot product over all attention outputs, 3) the distance map between residues within the protein's 3D structure, with each point value given as the negative of the 3D distance between the two residue positions, 4) a deleterious mutation map taken from HGMD data displaying the locations of deleterious mutation sites. All four visuals for each of the two proteins are displayed in Figure S5B. We can see from these visuals that while MutFormer's attention maps for each example do not perfectly correspond to either the 3D distance map or deleterious mutation location map, presumably due to the model's need to consider other relationships simultaneously, there is still notable resemblance present. For instance, in PDB: 1B1C's rollout attention, we find a resemblance with the deleteriousness map, as both have bright bands roughly at positions 12, 48, 74, 120, and 160. We also observe some resemblance between the rollout attention of MutFormer for PDB: 1P4O and the 3D distance map, we roughly observe a bright rectangle from positions 175 to the end of the sequence, a broad band from positions 150 to 200 running the length and width of the rectangle, and a rough rectangle from positions 0 to 100.

Supplemental Figures

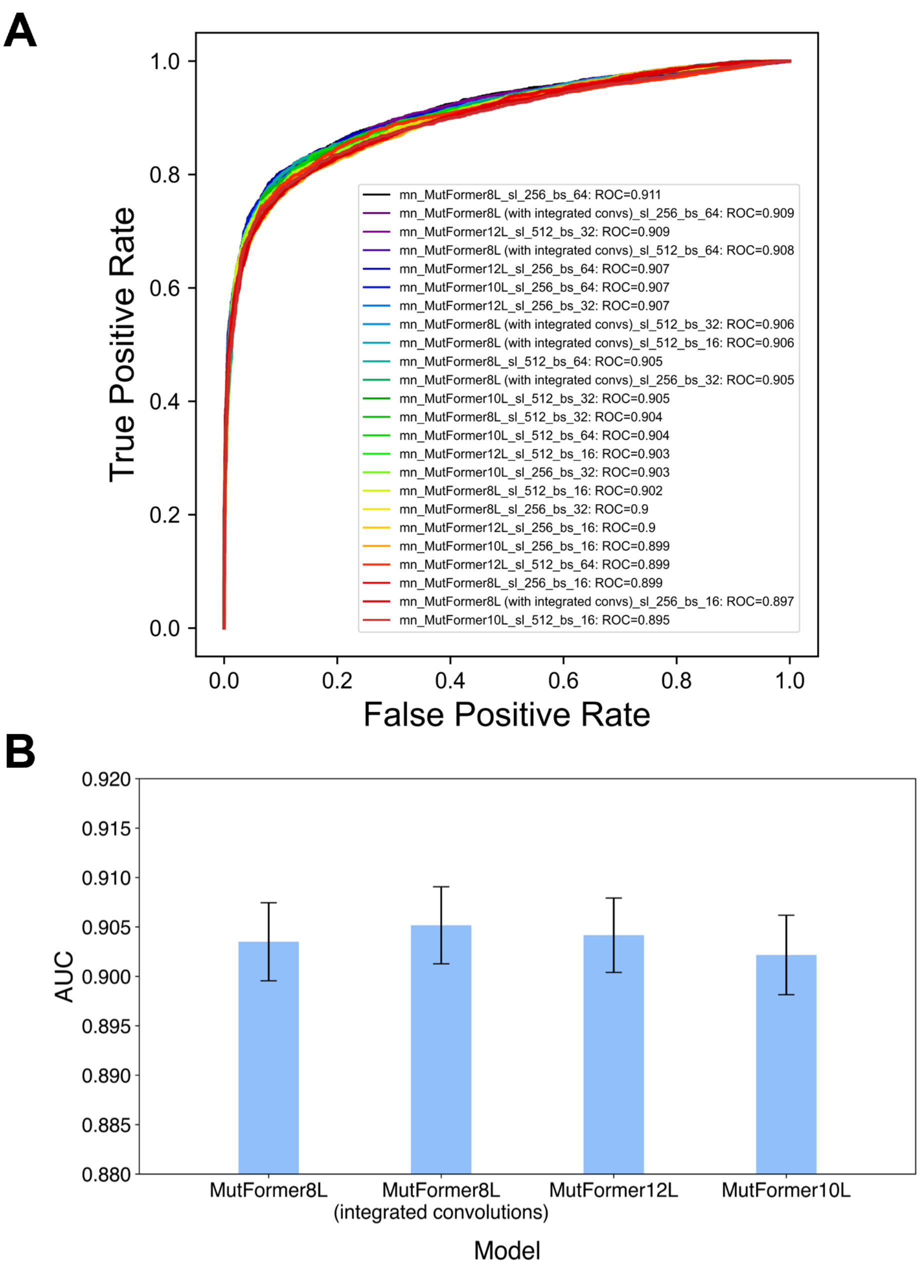

Figure S1

Fine-tuning internal comparison test 2: Performance comarison of MutFormer versus MutFormer *(with integrated convolutions).* **(A)** ROC curves for two different model architectures (class MutFormer and MutFormer (*with integrated convs*)) tested on varying sequence lengths and batch sizes. The labels are in the following format: "mn_[model name]_sl_[max input sequence length]_bs_[batch size]: ROC=[ROCAUC]". **(B)** Performance comparison of the four different models: MutFormer$_{8L}$*(with integrated convs)*, MutFormer$_{8L}$, MutFormer$_{10L}$, and MutFormer$_{12L}$.

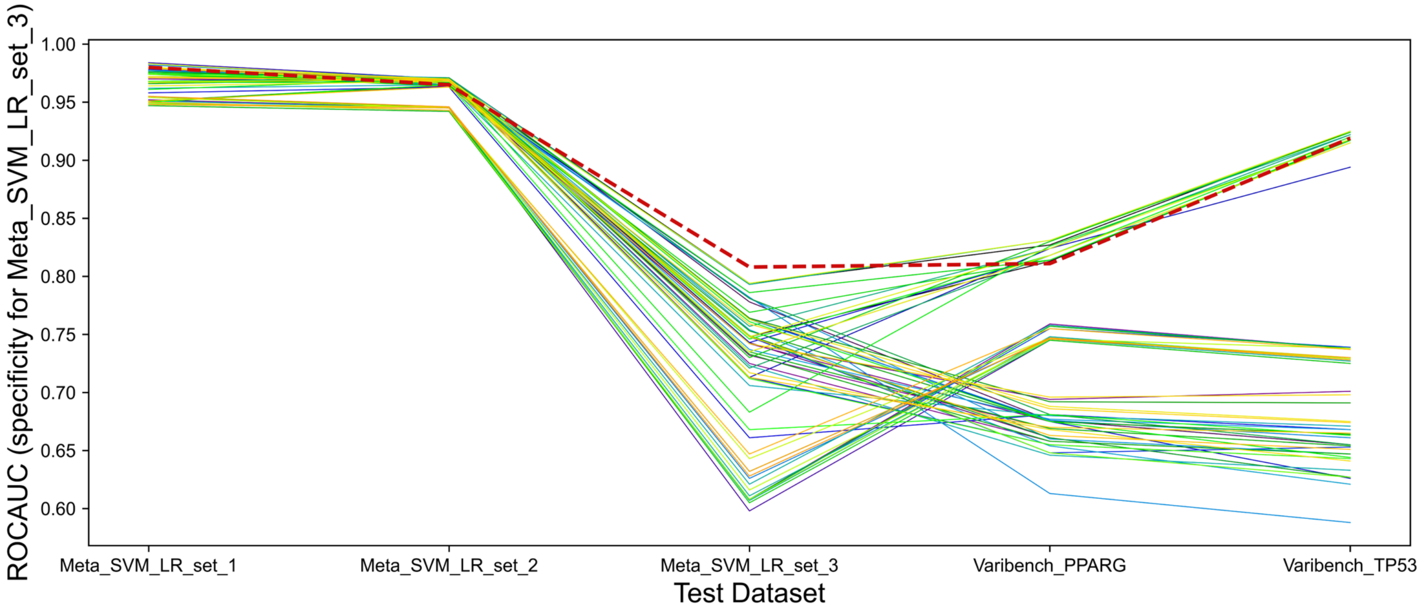

Figure S2

Summary of all testing dataset runs for MutFrmer (with external predictions). All MutFormer test runs for varying levels of "fit" are displayed as solid lines; the chosen best performing overall run, which represented MutFormer in our comparison vs other existing methods, is bolded and dashed. Note that for dataset 3, which contains only negative examples, the y-coordinate in the graph corresponds to specificity instead of ROCAUC.

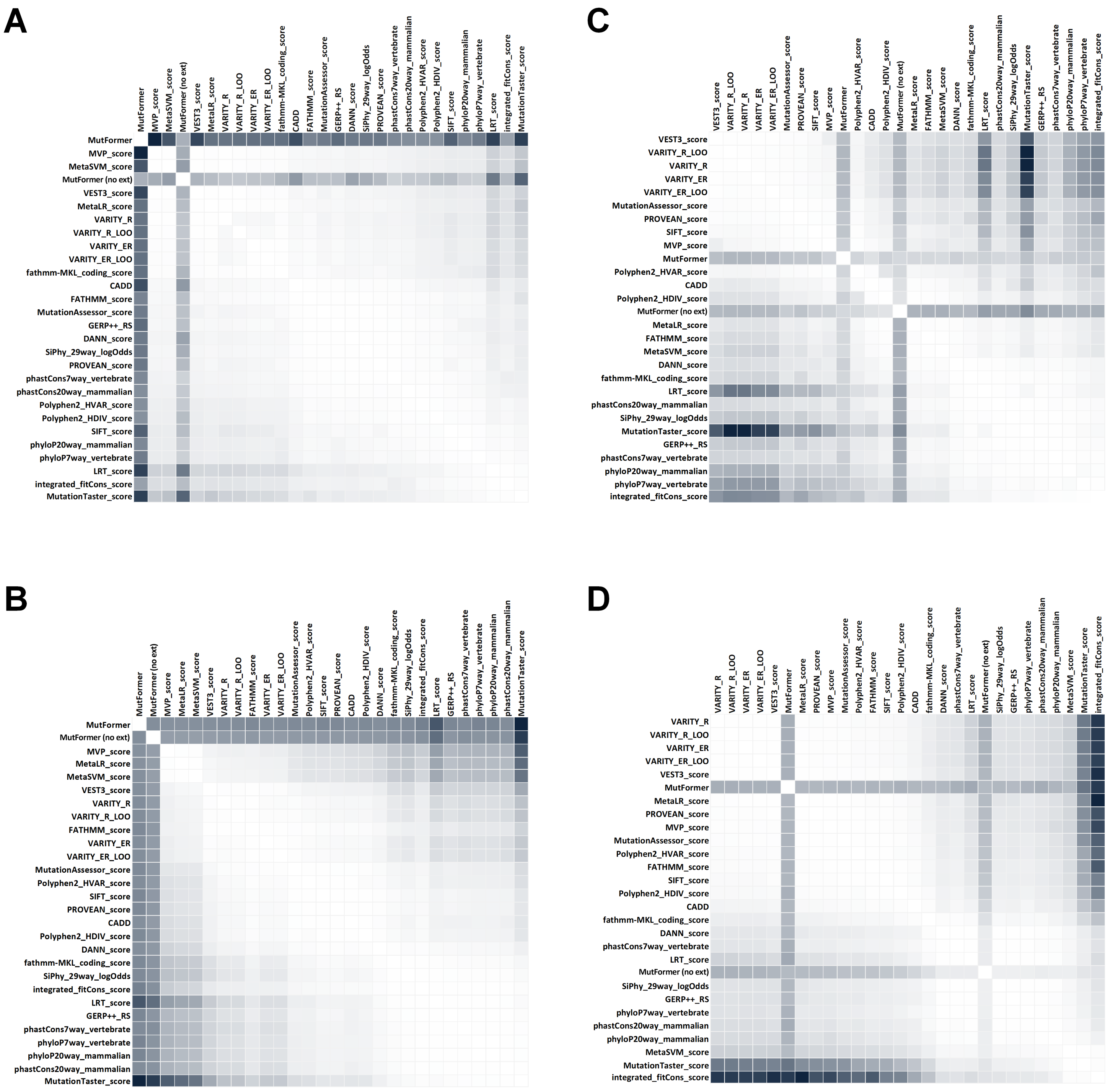

Figure S3

Delong test for MutFormer vs other methods: Delong test reporting statistical probability of equivalence between two ROC curves. Pixel values correspond to log scale probability of equilvalence (darker values indicate lower probability), and the ordering of the methods in the figure is based on their relative performance in each testing dataset. **(A)** Meta_SVM_LR_set_1, **(B)** MetaSVM_LR_set_2, **(C)** Varibench_PPARG, **(D)** Varibench_TP53.

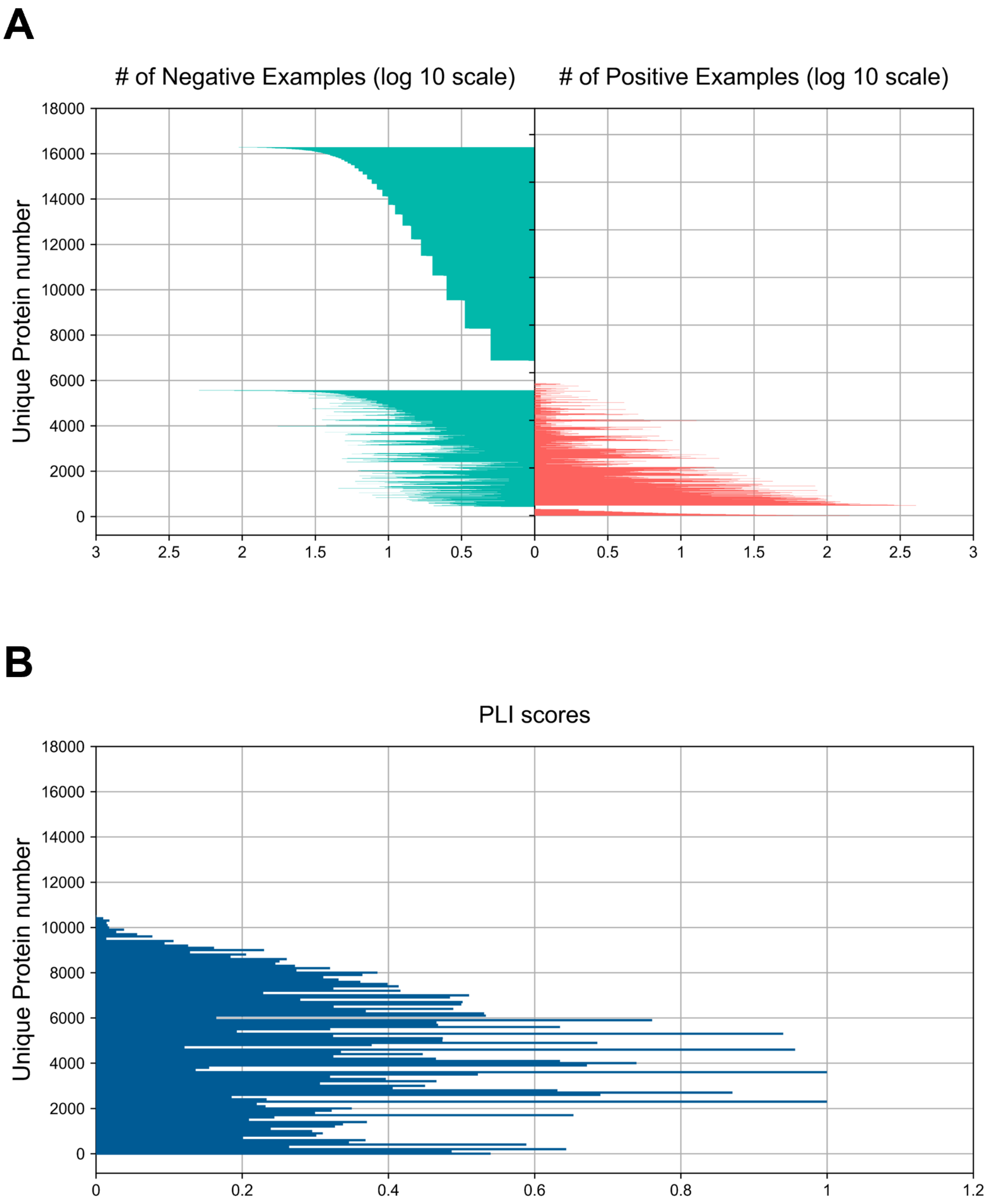

Figure S4

Per Protein Pathogenic and Benign Mutant Counts and Corresponding pLI Scores. Graphical display of the distributions of pathogenic and benign examples included in MutFormer's fine-tuning data at the protein level. Proteins are sorted primarily based on ratio of pathogenic examples to total examples, secondarily by number of pathogenic examples, and tertiarily inversely by the number of benign examples. **(A)** Counts of positive (pathogenic) and negative (benign) mutation examples for each protein ID within all examples present in MutFormer's finetune training data, **(B)** Loss of function intolerance probability (pLI) for each unique protein corresponding to part A based on GnomAD data.

A

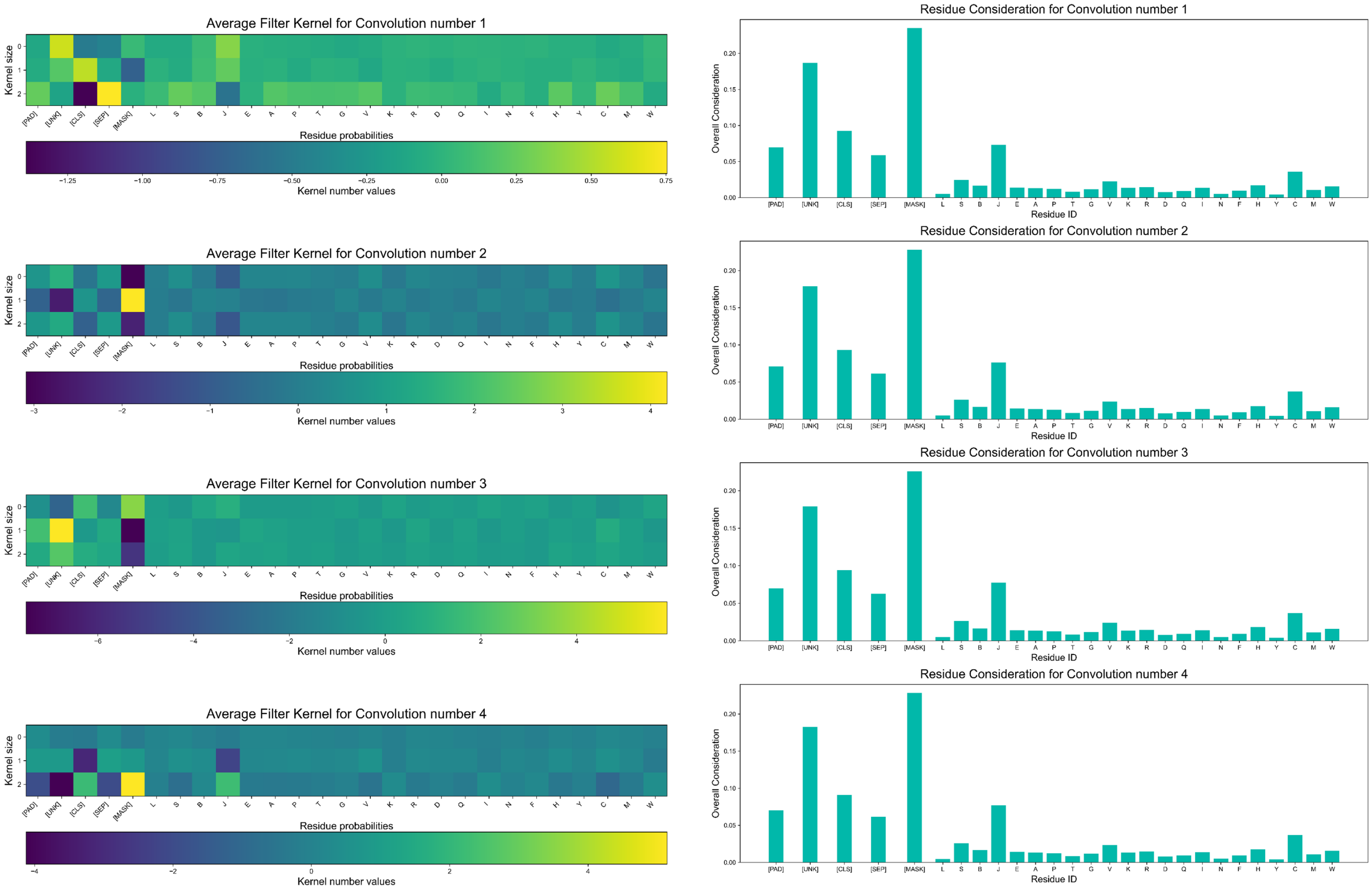
Average Filter Kernel for Convolution number 1
Kernel size
Residue probabilities
Kernel number values
Average Filter Kernel for Convolution number 2
Average Filter Kernel for Convolution number 3
Average Filter Kernel for Convolution number 4
[PAD] [UNK] [CLS] [SEP] [MASK] L S B J E A P T G V K R D Q I N F H Y C M W
Residue Consideration for Convolution number 1
Residue Consideration for Convolution number 2
Residue Consideration for Convolution number 3
Residue Consideration for Convolution number 4
Overall Consideration
Residue ID

B

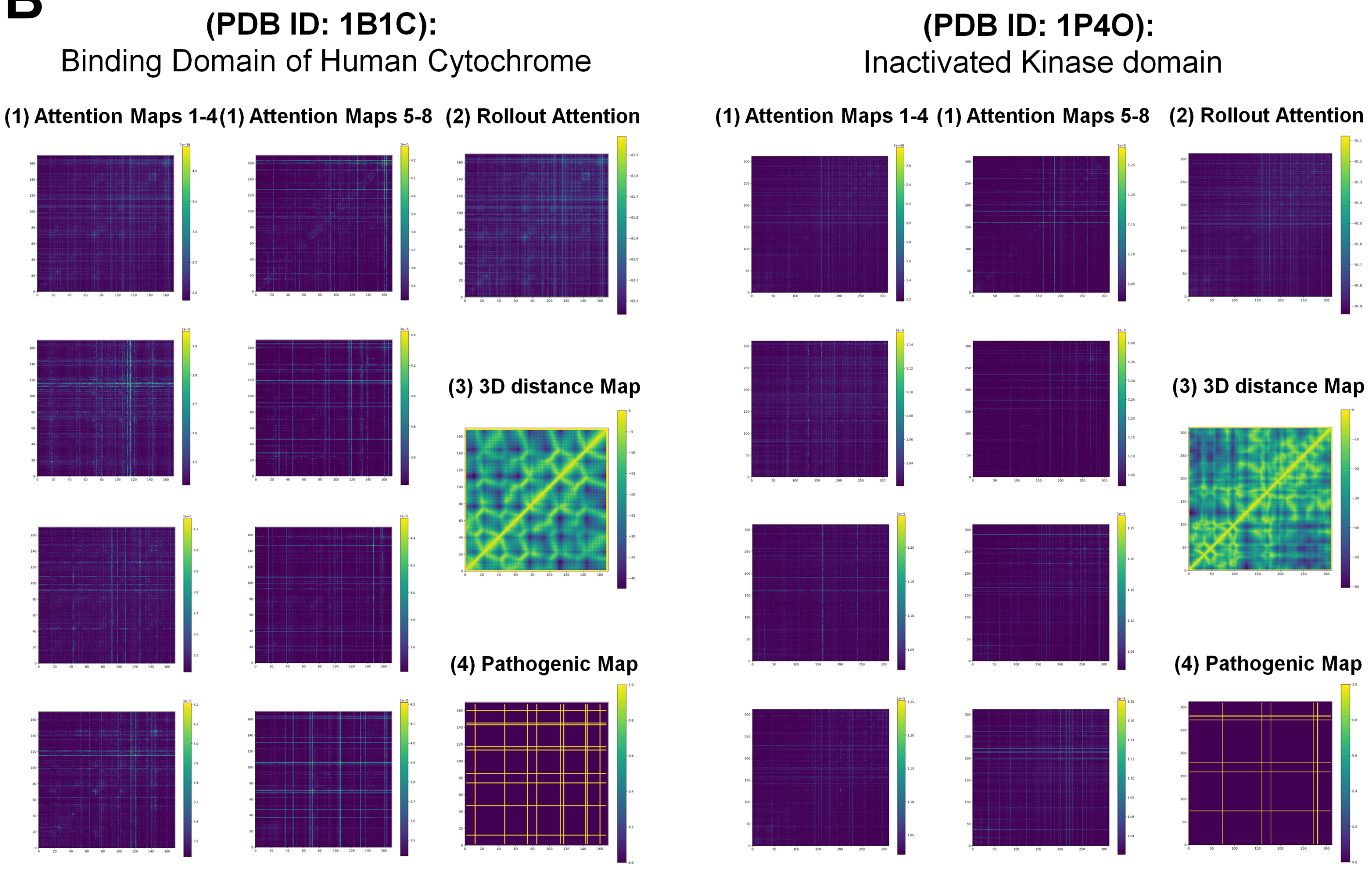
(PDB ID: 1B1C):
Binding Domain of Human Cytochrome
(1) Attention Maps 1-4
(1) Attention Maps 5-8
(2) Rollout Attention
(3) 3D distance Map
(4) Pathogenic Map
(PDB ID: 1P4O):
Inactivated Kinase domain
(1) Attention Maps 1-4
(1) Attention Maps 5-8
(2) Rollout Attention
(3) 3D distance Map
(4) Pathogenic Map

Figure S5

Model Weights Analysis: Graphical displays of an analysis of Mutformer's attention weights. **(A)** Average convolution filter (left) and overall residue consideration (right) for each of 4 convolutions used by Mutformer, **(B)** Attention weights analysis ((1) attention map for each attention layer, (2) rollout attention: dot producted attention map for all attention layers, (3) 3D distance map for the protein's true 3D structure, and (4) pathogenic map displaying all pathogenic locations found for each protein in the finetuning dataset) for two proteins: PDB: 1B1C (left) and PDB: 1P4O (right).

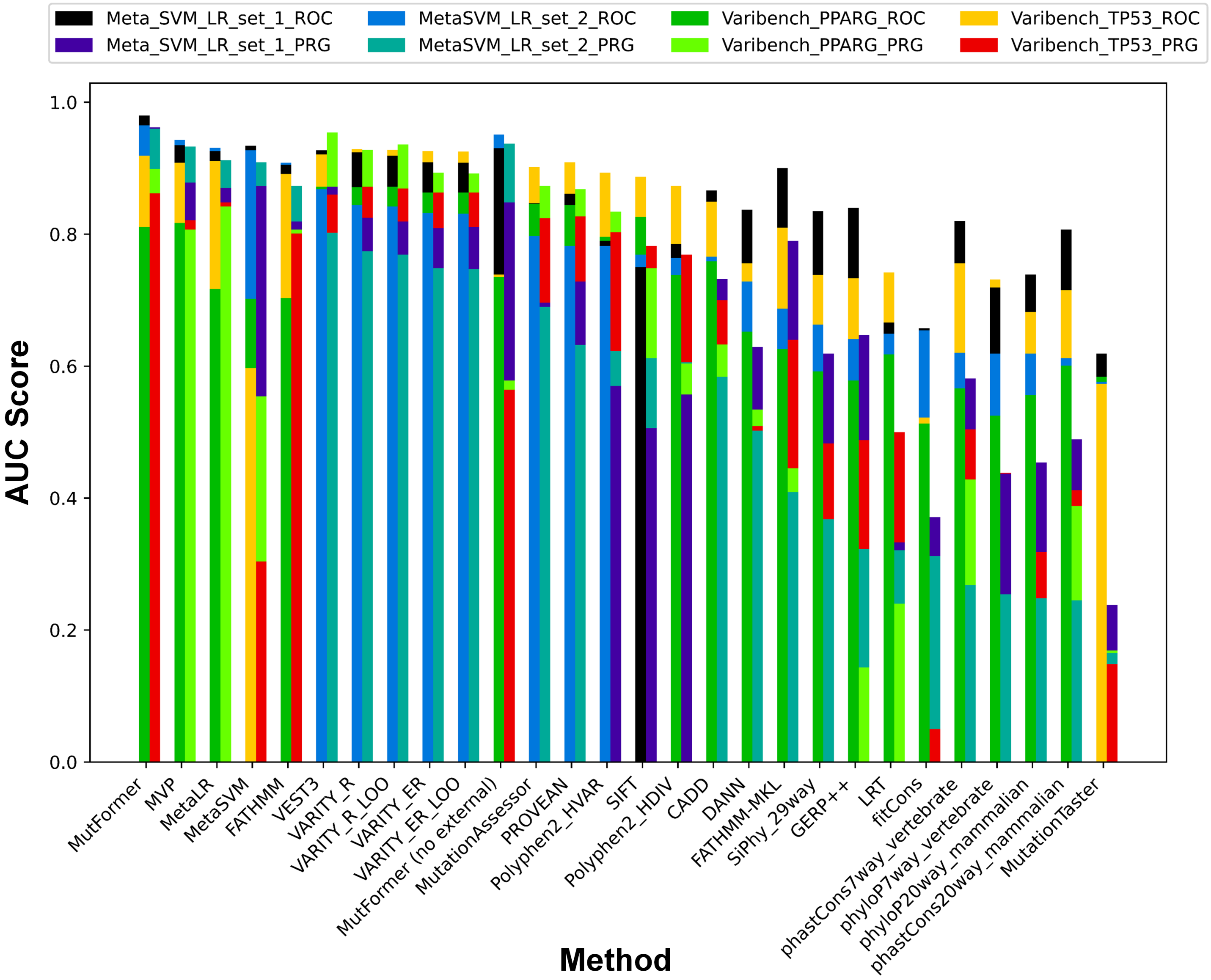

Figure S6

Bar Plot representation of Receiver Operator Characteristic and Precision Recall Gain AUCs for comparison with existing methods. Side by side bar graphs of each method of deleteriousness prediction for their performance as displayed in Figure 4 and 5. For each method, the left bar displays the ROC AUC of that method's performance on datasets 1, 2, 4, and 5. The right bar displays PRG AUC. Note that PRG AUC values that were below 0 were clipped to 0.

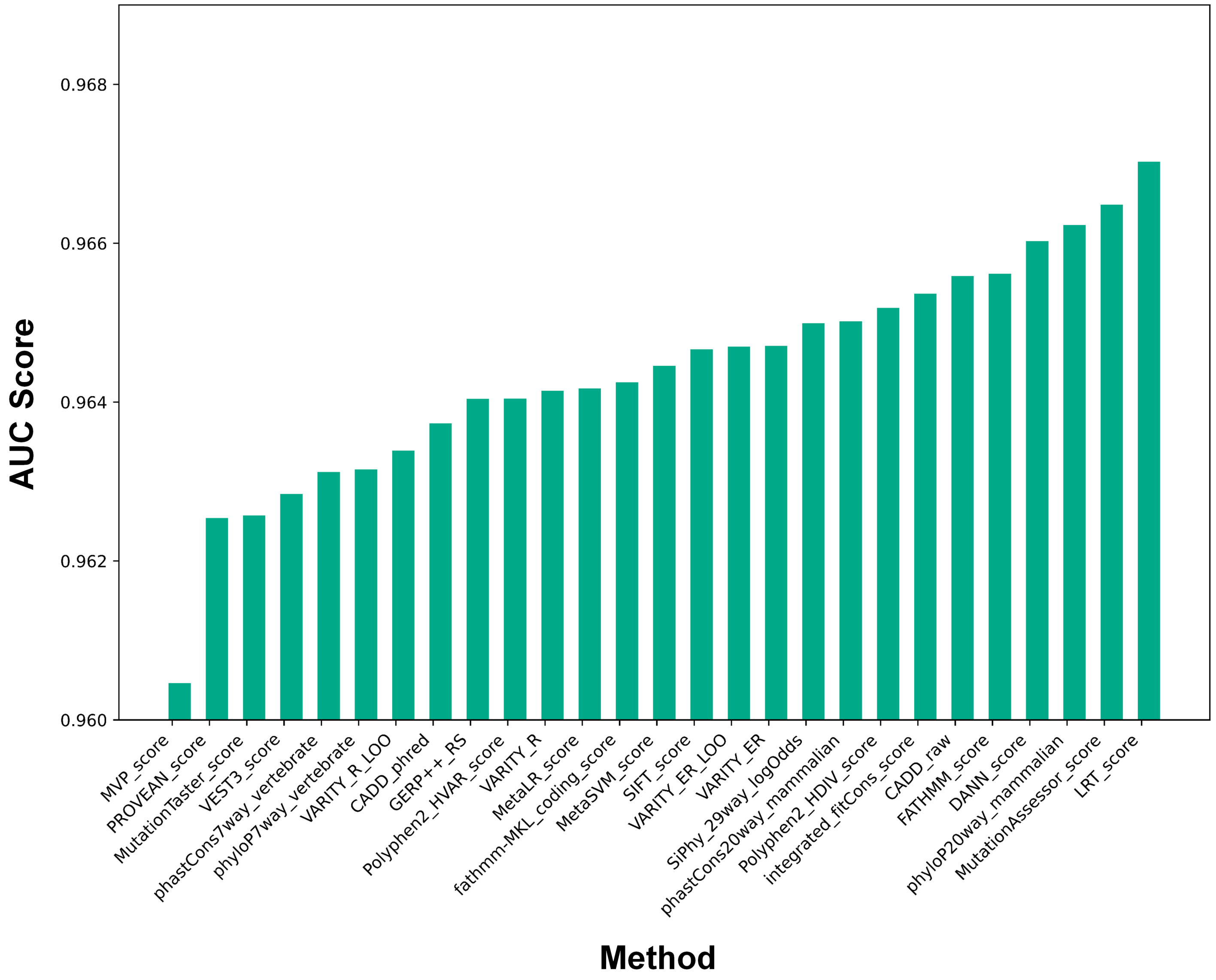

Figure S7

Ablation study results for MutFormer's use of external predictions. ROC AUC performance of MutFormer on our independent validation dataset after removing each external prediction (for each external prediction, all other predictions were kept while all entries for that prediction were set to null). The MutFormer model represented here is MutFormer_{8L} with integrated convolutions, finetuned on a batch size of 32 with 0 freezing layers.

Supplemental Tables

Table S1

Hyperparameters and training description during pretraining for each model.

Model Name	Learning Rate Decay per Step	Batch Size	Steps per Epoch	Total Steps	Training Time
$MutBERT_{8L}$	1.33e-11	64	2300	1.5M	~130 hrs
$MutBERT_{10L}$	1.33e-11	64	2300	1.5M	~160 hrs
$MutFormer_{8L}$	1.33e-11	64	2300	1.5M	~150 hrs
$MutFormer_{10L}$	1.33e-11	64	2300	1.5M	~175 hrs
$MutFormer_{12L}$	1.00e-11	32	4600	2.0M	~275 hrs
$MutFormer_{8L}$ *(with integrated convs)*	1.33e-11	64	2300	1.5M	~150 hrs

Additional hyperparameters constant for all models:

- Initial Learning Rate: 2e-5
- Weight Decay (For Adam Optimizer): 0.01
- Gradient Clip amount (During Optimization): 1.0

Table S2

Loss and accuracy on the pretraining task (masked residue prediction).

Model Name	Training split		Test split	
	Loss	Accuracy	Loss	Accuracy
$MutBERT_{8L}$	1.4641	0.5538	2.0297	0.4021
$MutBERT_{10L}$	1.1360	0.6504	1.7248	0.4863
$MutFormer_{8L}$	0.9872	0.6984	1.2274	0.6212
$MutFormer_{10L}$	0.8560	0.7384	1.0961	0.6631
$MutFormer_{12L}$	0.8338	0.7460	1.0727	0.6730
$MutFormer_{8L}$ *(with integrated convs)*	0.8305	0.7475	1.0590	0.6930

Table S3

The numbers of SNVs in the test sets that were missing from each method.

Method	Reference	Number of missing SNVs					
		Set 1	Set 2	Set 3	Set 4	Set 5	Set 6
MutFormer	-	0	0	0	0	0	0
SIFT	5	16	2	203	72	102	104
PolyPhen2-HDIV	6	5	0	100	22	21	15
PolyPhen2-HVAR	6	5	0	100	22	21	15
LRT	7	130	10	701	22	501	256
MutationTaster	8	8	0	65	25	14	14
MutationAssessor	9	27	2	228	23	64	69
FATHMM	10	142	2	484	50	186	207
PROVEAN	11	12	1	160	67	46	51
VEST3	12	1	0	74	22	13	13
CADD	13,14	0	0	46	22	0	0
DANN	15	0	0	46	22	0	0
FATHMM-MKL	16	0	0	46	22	0	0
MetaSVM	17	1	0	46	22	3	3
MetaLR	17	1	0	46	22	3	3
fitCons	18	87	3	1195	159	433	433
GERP++	19	0	0	50	22	7	7
PhyloP-7way-vertebrate	20	0	0	47	22	1	1
PhyloP-20way-mammalian	20	0	0	47	22	0	0
PhastCons-7way-vertebrate	21	0	0	47	22	1	1
PhastCons-20way-mammalian	21	0	0	47	22	0	0
SiPhy-29way (log odds)	22	2	0	68	22	12	9
VARITY_ER	23	95	6	516	77	428	200
VARITY_ER_LOO	23	95	6	516	77	428	200
VARITY_R	23	95	6	516	77	428	200
VARITY_R_LOO	23	95	6	516	77	428	200
MVP	24	0	4	969	6	228	171

Note: the total numbers of SNVs in the test sets are outlined in Table 2.

Table S4

The ROC (Receiver Operator Characteristic) and RPG (Precision- Recall- Gain) score of various methods on the testing dataset. This table is provided as a separate CSV file due to the presence of many columns.

Table S5

ProteinGym Evaluation results between MutFormer and other methods included in ProteinGym.

Method	ROC AUC Score	Matthews Correlation Coefficient	Spearman Rank Correlation
Ensemble Tranception & EVE	0.756	0.35	0.435
ESM-1v (ensemble)	0.751	0.341	0.418
Tranception M	0.745	0.339	0.415
Tranception L	0.744	0.335	0.413
EVE (ensemble)	0.738	0.334	0.403
Tranception S	0.735	0.329	0.398
DeepSequence (ensemble)	0.735	0.326	0.396
EVE (single)	0.735	0.324	0.395
Progen2 (ensemble)	0.733	0.322	0.394
ESM-1v (single)	0.732	0.322	0.39
DeepSequence (single)	0.732	0.321	0.39
MSA Transformer (ensemble)	0.729	0.317	0.39
Progen2 Base	0.729	0.316	0.379
Wavenet	0.728	0.315	0.379
EVmutation	0.725	0.313	0.378
MSA Transformer (single)	0.724	0.311	0.376
Progen2 M	0.724	0.306	0.376
Mutformer	0.723	0.305	0.376
Progen2 L	0.723	0.304	0.375
RITA (ensemble)	0.723	0.301	0.373
Progen2 S	0.717	0.3	0.367
RITA L	0.717	0.298	0.362
Tranception L no retrieval	0.713	0.296	0.359
Site-Independent	0.712	0.294	0.358
RITA M	0.711	0.288	0.354
RITA XL	0.709	0.284	0.348
Progen2 XL	0.702	0.271	0.338
RITA S	0.682	0.245	0.306

References

1. Karczewski, K.J., Francioli, L.C., Tiao, G., et al. (2020). The mutational constraint spectrum quantified from variation in 141,456 humans. Nature ***581***, 434-443.
2. Stenson, P.D., Mort, M., Ball, E.V., et al. (2020). The Human Gene Mutation Database (HGMD((R))): optimizing its use in a clinical diagnostic or research setting. Hum Genet ***139***, 1197-1207.
3. Frazer, J., Notin, P., Dias, M., et al. (2021). Disease variant prediction with deep generative models of evolutionary data. Nature ***599***, 91-95.
4. Notin, P., Dias, M., Frazer, J., et al. (2022). Tranception: protein fitness prediction with autoregressive transformers and inference-time retrieval. International Conference on Machine Learning. PMLR.
5. Ng, P.C., and Henikoff, S. (2003). SIFT: Predicting amino acid changes that affect protein function. Nucleic Acids Res ***31***, 3812-3814.
6. Adzhubei, I.A., Schmidt, S., Peshkin, L., et al. (2010). A method and server for predicting damaging missense mutations. Nat Methods ***7***, 248-249.
7. Chun, S., and Fay, J.C. (2009). Identification of deleterious mutations within three human genomes. Genome Res ***19***, 1553-1561.

8. Schwarz, J.M., Cooper, D.N., Schuelke, M., and Seelow, D. (2014). MutationTaster2: mutation prediction for the deep-sequencing age. Nat Methods ***11***, 361-362.
9. Reva, B., Antipin, Y., and Sander, C. (2011). Predicting the functional impact of protein mutations: application to cancer genomics. Nucleic Acids Res ***39***, e118.
10. Shihab, H.A., Gough, J., Cooper, D.N., et al. (2013). Predicting the functional, molecular, and phenotypic consequences of amino acid substitutions using hidden Markov models. Hum Mutat ***34***, 57-65.
11. Choi, Y., Sims, G.E., Murphy, S., et al. (2012). Predicting the functional effect of amino acid substitutions and indels. PLoS One ***7***, e46688.
12. Carter, H., Douville, C., Stenson, P.D., et al. (2013). Identifying Mendelian disease genes with the variant effect scoring tool. BMC Genomics ***14 Suppl 3***, S3.
13. Rentzsch, P., Witten, D., Cooper, G.M., et al. (2019). CADD: predicting the deleteriousness of variants throughout the human genome. Nucleic Acids Res ***47***, D886-D894.
14. Kircher, M., Witten, D.M., Jain, P., et al. (2014). A general framework for estimating the relative pathogenicity of human genetic variants. Nat Genet ***46***, 310-315.
15. Quang, D., Chen, Y., and Xie, X. (2015). DANN: a deep learning approach for annotating the pathogenicity of genetic variants. Bioinformatics ***31***, 761-763.
16. Shihab, H.A., Rogers, M.F., Gough, J., et al. (2015). An integrative approach to predicting the functional effects of non-coding and coding sequence variation. Bioinformatics ***31***, 1536-1543.
17. Dong, C., Wei, P., Jian, X., et al. (2015). Comparison and integration of deleteriousness prediction methods for nonsynonymous SNVs in whole exome sequencing studies. Hum Mol Genet ***24***, 2125-2137.
18. Gulko, B., Hubisz, M.J., Gronau, I., and Siepel, A. (2015). A method for calculating probabilities of fitness consequences for point mutations across the human genome. Nat Genet ***47***, 276-283.
19. Davydov, E.V., Goode, D.L., Sirota, M., et al. (2010). Identifying a high fraction of the human genome to be under selective constraint using GERP++. PLoS Comput Biol ***6***, e1001025.
20. Pollard, K.S., Hubisz, M.J., Rosenbloom, K.R., and Siepel, A. (2010). Detection of nonneutral substitution rates on mammalian phylogenies. Genome Res ***20***, 110-121.
21. Siepel, A., Bejerano, G., Pedersen, J.S., et al. (2005). Evolutionarily conserved elements in vertebrate, insect, worm, and yeast genomes. Genome Res ***15***, 1034-1050.
22. Garber, M., Guttman, M., Clamp, M., et al. (2009). Identifying novel constrained elements by exploiting biased substitution patterns. Bioinformatics ***25***, i54-62.
23. Wu, Y., Li, R., Sun, S., et al. (2021). Improved pathogenicity prediction for rare human missense variants. Am J Hum Genet.
24. Qi, H., Zhang, H., Zhao, Y., et al. (2021). MVP predicts the pathogenicity of missense variants by deep learning. Nat Commun ***12***, 510.
